# Simultaneous induction of vasculature and neuronal network formation on a chip reveals a dynamic interrelationship between cell types

**DOI:** 10.1186/s12964-023-01159-4

**Published:** 2023-06-14

**Authors:** Lotta Isosaari, Hanna Vuorenpää, Alma Yrjänäinen, Fikret Emre Kapucu, Minna Kelloniemi, Toni-Karri Pakarinen, Susanna Miettinen, Susanna Narkilahti

**Affiliations:** 1 NeuroGroup, Faculty of Medicine and Health Technology, Tampere University, Tampere, Finland; 2 Adult Stem Cell Group, Faculty of Medicine and Health Technology, Tampere University, Tampere, Finland; 3 Research, Development and Innovation Centre, Tampere University Hospital, Tampere, Finland; 4 Department of Plastic and Reconstructive Surgery, Tampere University Hospital, Tampere, Finland; 5 Regea Cell and Tissue Center, Tampere University, Tampere, Finland

**Keywords:** Angiogenesis, Cellular communication, Human cells, Microfluidic, Neurovascular interactions, 3D cell culture

## Abstract

**Background:**

Neuronal networks receive and deliver information to regulate bodily functions while the vascular network provides oxygen, nutrients, and signaling molecules to tissues. Neurovascular interactions are vital for both tissue development and maintaining homeostasis in adulthood; these two network systems align and reciprocally communicate with one another. Although communication between network systems has been acknowledged, the lack of relevant in vitro models has hindered research at the mechanistic level. For example, the current used in vitro neurovascular models are typically established to be short-term (≤ 7 days) culture models, and they miss the supporting vascular mural cells.

**Methods:**

In this study, we utilized human induced pluripotent stem cell (hiPSC) -derived neurons, fluorescence tagged human umbilical vein endothelial cells (HUVECs), and either human bone marrow or adipose stem/stromal cells (BMSCs or ASCs) as the mural cell types to create a novel 3D neurovascular network-on-a-chip model. Collagen 1–fibrin matrix was used to establish long-term (≥ 14 days) 3D cell culture in a perfusable microphysiological environment.

**Results:**

Aprotinin-supplemented endothelial cell growth medium-2 (EGM-2) supported the simultaneous formation of neuronal networks, vascular structures, mural cell differentiation, and the stability of the 3D matrix. The formed neuronal and vascular networks were morphologically and functionally characterized. Neuronal networks supported vasculature formation based on direct cell contacts and by dramatically increasing the secretion of angiogenesis-related factors in multicultures in contrast to cocultures without neurons. Both utilized mural cell types supported the formation of neurovascular networks; however, the BMSCs seemed to boost neurovascular networks to greater extent.

**Conclusions:**

Overall, our study provides a novel human neurovascular network model that is applicable for creating in vivo-like tissue models with intrinsic neurovascular interactions. The 3D neurovascular network model on chip forms an initial platform for the development of vascularized and innervated organ-on-chip and further body-on-chip concepts and offers the possibility for mechanistic studies on neurovascular communication both under healthy and in disease conditions.

Video Abstract

**Supplementary Information:**

The online version contains supplementary material available at 10.1186/s12964-023-01159-4.

## Background

In humans, most tissues and organs depend on neuronal and vascular networks to maintain their normal functions and homeostasis. Neuronal networks function by receiving and delivering information to regulate bodily functions whereas vascular networks provide oxygen, nutrients, and signaling molecules to tissues. Increasing evidence shows that crosstalk between network systems and neurovascular cell interactions are crucial in development, tissue repair processes and homeostasis [1, 2].

Nerves and vessels have been suggested to depend on each other during tissue formation in development. Nerves and blood vessels respond to the same guidance molecules, such as semaphorins, ephrins, vascular endothelial growth factor (VEGF), and endothelin, emphasizing the concordant developmental processes [3, 4]. During development, the patterning of nerves and blood vessels is orchestrated to drive their close anatomical apposition to each other [5]. For example, in the adult brain, neurons are located within 15 µm of the closest blood vessel [6] to fulfill the metabolic needs of the nerves related to the blood supply, and in turn, enable the control of blood vessel tone by nerves [7]. The copatterning, functional properties, and responsiveness to similar guidance molecules indicate that these two network systems form functionally integrated networks where neuronal activity and vascular dynamics are closely coupled [7]. The complex cellular and molecular interactions and mechanisms by which the network systems affect each other are, however, poorly understood [1]. Given their tight interrelationship, it is not surprising that disruptions in one network can provoke dysfunction in the other network [8]. Disruption of these interactions has been associated with neurodegenerative diseases and nervous system disorders, e.g., multiple sclerosis (MS) [9], diabetic neuropathy [10] and vascular dementia [11].

Organ-on-chip (OoC) models consisting of multiple human cell types are essential for studying cell type-specific contributions to the interactions between neuronal and vascular networks. Blood vessels consist mainly of vascular endothelial cells (ECs) which form the inner vessel wall, and supporting mural cells, that is, pericytes and smooth muscle cells, which align and support the forming vessels. To recapitulate physiological functions, both ECs and mural cells need to be included in OoC models, as mural cells are important elements of functional vasculature [12]. Mural cells also participate in the neurovascular system by interacting with both ECs and neurons; for example, they participate in the regulation of cerebral blood flow by responding to stimuli from neuronal and glial cells [13]. The lack of mural cells may prevent true disease modeling as pericyte activation has been identified as a critical part of the pathophysiology of central nervous system (CNS) diseases [14]. Adult mesenchymal stem/stromal cells (MSCs) have been successfully used as sources for mural cells in in vitro 3D vascular models [15, 16]. MSCs, such as bone marrow-derived MSCs (BMSCs) and adipose tissue-derived MSCs (ASCs) are attractive sources for mural cells due to their availability and natural location near blood vessels [17].

OoC modeling of integrated neuronal and vascular networks has remained a challenge, as these two network systems form complex 3D structures, require long-term coculturing to mature, and need to be cultured in medium suitable for multiple cell types. Many of the models have been established in 2D format, on the basis of, e.g., Transwell approaches [18–21], or via direct cocultures of neurons and ECs [20, 22] mainly to model blood‒brain barrier (BBB) functions. However, establishing models in a 3D environment is essential for the formation of in vivo* -*like networks, interactions, and connections between cell types [23, 24]. The formation of neuronal networks has been reported in various static 3D scaffolds [25–27] whereas 3D vasculature models have been more frequently established on active microfluidic platforms [16, 28], which provide physiological mechanical stimuli to induce vessel formation and enable the delivery of nutrients and oxygen [29]. Few 3D neurovascular network models in microfluidic platforms exist, e.g., for motor neuron [30] and BBB applications [31, 32]. Typically, these models lack supporting mural cell types and/or are cultured short-term (≤ 7 days) which limits the time for interaction between the two network systems. Additionally, the codependence and interactions between neuronal and vascular networks have not been addressed in detail in current models. There are also examples of integration of brain organoids in vivo into rodent brain enabling vascularization of the organoid by host tissue [33].

In this study, we established a long-term 3D human cell-based neurovascular model on a microfluidic chip. We investigated neuronal and vascular network formation and mural cell differentiation in different cell culture media and found conditions that supported all the cell types in the multicultures. Using angiogenic medium, we were able to establish a reproducible long-term 3D model with consistent neuronal and vascular networks. Vasculature formation was stable on the presence of an integrated neuronal network when both mural cell types were used. Moreover, the angiogenic process was promoted in the presence of the neuronal networks, as indicated by protein secretion analysis. Neuronal maturation was evident both morphologically and functionally in the multicultures. Our results suggest that neurons play an inducing role in angiogenesis and highlight the role of neurovascular interactions in the dual development of both network systems. The presented novel model can be utilized to study neurovascular interactions during organ development and in disease states. It paves the way for the development of innervated OoC models based on the body-on-chip (BoC) concept.

## Methods

### Cell culture

#### Neuronal differentiation

The UTA.04511.WTs human induced pluripotent stem cell (hiPSC) line [34] was derived in house and used for neuronal differentiation. The line was derived at the Faculty of Medicine and Health Technology (MET), iPS Cells facility, Tampere University, Finland. The hPSCs used in this study were acquired from voluntary subject who had given written and informed consent. The project has a supportive statement from Ethics Committee of the Expert Responsibility area of Tampere University Hospital to use the named hPSC line in neuronal research (R20159). The pluripotency of the line was confirmed regularly, and all cultures maintained normal karyotypes and were free of mycoplasma. The hiPSCs were expanded and differentiated into cortical neurons in a feeder-free culture as previously described [35, 36]. The differentiation included neuronal induction, precursor expansion, and maturation phases. Day 32 or 46 of differentiation, the cells were plated for experiments.

#### Endothelial cells

Pooled human umbilical vein endothelial cells (HUVECs) expressing green fluorescent protein (GFP, CellWorks) or red fluorescent protein (RFP, Angioproteomie) were used. GFP- and RFP-HUVECs were cultured with a Endothelial Cell Growth Medium-2 Bullet Kit (EGM-2, Lonza) consisting of Endothelial Cell Growth Basal Medium (EBM-2) and EGM-2 Supplements. However, we did not use the fetal bovine serum supplied with the kit; in contrast, 2% human serum (HS, BioWest) was used. The cells were used between Passages 4 and 6.

#### Human adipose stem/stromal cells

Human adipose stem/stromal cells (ASCs) were isolated from subcutaneous abdominal tissue samples obtained from two donors (Supplementary Table 1). The tissue samples were obtained at the Tampere University Hospital Department of Plastic Surgery with each donor’s written informed consent and processed with the ethical approval of the Ethics Committee of the Expert Responsibility area of Tampere University Hospital (R15161). The cells were isolated as described previously [37]. The ASCs were cultured in α-modified minimum essential medium (α-MEM, Gibco) supplemented with 5% HS, 100 U/ml penicillin, and 100 µg/ml streptomycin (Pen/Strep, Lonza) and used between Passages 3 and 5. The mesenchymal origin of the ASCs was confirmed by surface marker expression analysis with flow cytometry and assessment of their adipogenic and osteogenic differentiation potential (Supplementary Table 2).

#### Human bone marrow -derived stem/stromal cells

Human bone marrow -derived stem/stromal cells (BMSCs) were isolated from bone marrow samples from two donors undergoing orthopedic surgery at the Tampere University Hospital Department of Orthopedics and Traumatology (Supplementary Table 1). The bone marrow samples were obtained with each donor’s written informed consent and processed with the ethical approval of the Ethics Committee of the Expert Responsibility area of Tampere University Hospital, Tampere, Finland (R15174). The cells were isolated as described previously [38]. The BMSCs were cultured in α-MEM supplemented with 5% HS, 100 U/ml/100 µg/ml Pen/Strep, and 5 ng/ml basic fibroblast growth factor (FGF-B, Miltenyi Biotec) and used between Passages 5 and 6. The mesenchymal origin of BMSCs was confirmed by surface marker expression analysis with flow cytometry and assessment of their adipogenic and osteogenic differentiation potential (Supplementary Table 2).

### Formation of neurovascular networks on a microfluidic chip

**Fig. 1:**
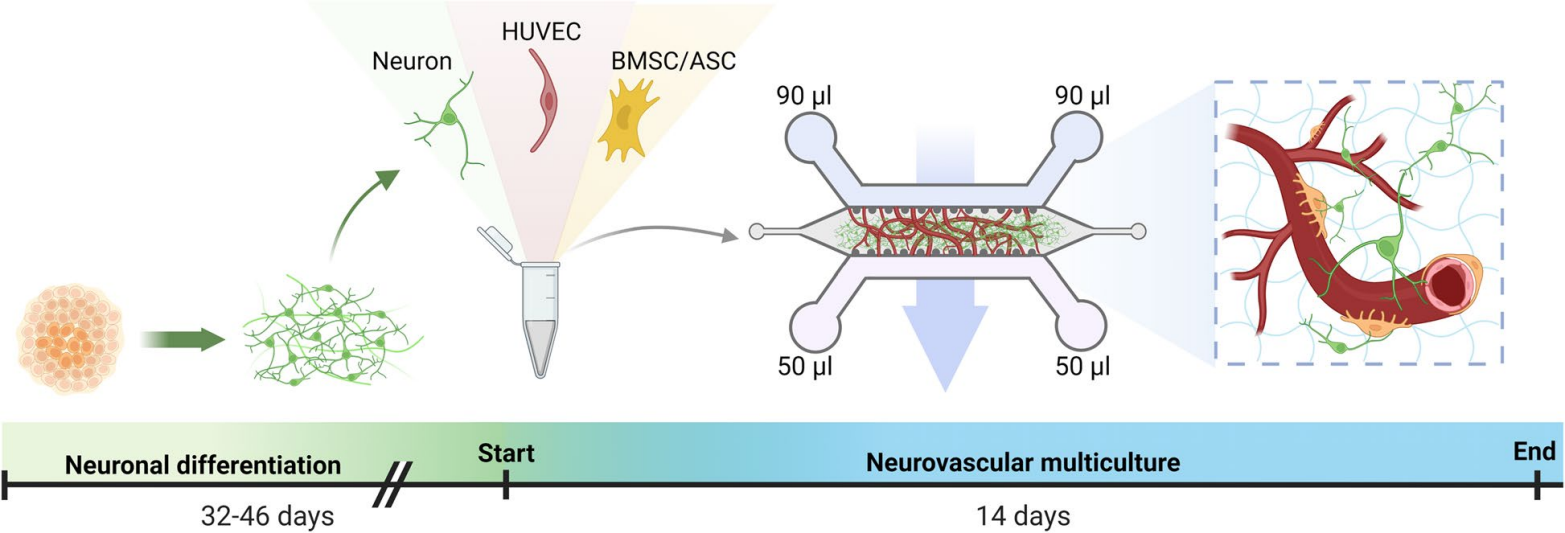
Experimental timeline for the formation of the 3D neurovascular networks on a chip. Created with BioRender.com

To form 3D neurovascular networks on a chip (hereafter referred to as “multicultures”), the cells were cultured in a gel mixture composed at the final concentrations of 1 mg/ml rat tail collagen 1 (Gibco), 1.5 mg/ml human fibrinogen (Lonza) and 0.6 iU/ml thrombin (Sigma‒Aldrich). GFP- or RFP-HUVECs were embedded at a density of 5.0 × 10^6^ cells/ml, neurons were embedded at 2.5 × 10^6^ cells/ml and BMSCs or ASCs were embedded at density of 1.0 × 10^6^ cells/ml in 10 μl the collagen 1–fibrin gel and injected into the hydrogel channel of microfluidic chip (DAX1, AIM Biotech, Fig. 1). The gel was injected immediately after thrombin and fibrinogen were mixed together to start gelation and was allowed to polymerize in a humidified chamber for 30 min at 37 °C before medium was added. Different cell culture media were tested to find the optimal culturing conditions for the multicultures. The tested media included neural maturation medium (NMM) consisting of a 50:50 mixture of D-MEM/F12 (with GlutaMAX) and neurobasal medium, 0.5% N2, 1% B27 with retinoic acid, 0.5 mM GlutaMAX, 0.5% NEEA, 50 µM 2-mercaptoethanol, 0.1% Pen/Strep (all from Thermo Fisher Scientific), 2.5 µg/mL insulin (Sigma‒Aldrich), 20 ng/mL brain-derived neurotrophic factor (BDNF, R&D Systems) 10 ng/mL glial-derived neurotrophic factor (GDNF, R&D Systems), 500 µM dibutyryl-cyclic AMP (db-cAMP, Sigma‒Aldrich), and 200 µM ascorbic acid (AA, Sigma‒Aldrich). A ROCK inhibitor (10 µM, Sigma‒Aldrich) was added to the NMM during cell seeding to support cell survival. Angiogenic EGM-2 and 50:50 EGM-2:NMM mixture were tested for use with the multicultures. According to preliminary testing, the shrinkage and degradation of the hydrogel was noticed in neuron samples with all media compositions (Supplementary Fig. 1). Thus, aprotinin 40 µg/ml (ab146286, Abcam) was added to medium in all media changes to prevent the degradation of fibrin. On the chip, a gravity-based perfusion was generated between the two medium channels: 90 µl of culture medium was added into both reservoirs of one medium channel and 50 µl of medium was added into both reservoirs of the other medium channel to generate interstitial flow through the hydrogel. This procedure was repeated daily when the medium was changed, and the flow direction was consistently maintained. The chips were cultured at + 37 °C in humidified incubator with 5% CO_2_. GFP- or RFP-HUVECs cultured together with BMSCs or ASCs without neurons (hereafter referred to as “cocultures”) and cultures consisting of only neurons (hereafter referred to as “monocultures”) were prepared and cultured at the same final cell densities (GFP- or RFP-HUVECs were embedded at a density of 5.0 × 10^6^ cells/ml, BMSCs or ASCs were embedded at density of 1.0 × 10^6^ cells/ml and neurons were embedded at 2.5 × 10^6^ cells/ml) in the same manner and served as the control for evaluating the neuronal impact and the effect of neurovascular interactions.

### Immunostaining

Samples on microfluidic chips were fixed with 4% paraformaldehyde in PBS for 30 min followed by two washes with PBS for 10 min each time at room temperature (RT). For permeabilization, the samples were treated with 0.3% Triton-X in PBS for 10 min and blocked thereafter with 10% normal donkey serum (NDS, Millipore), 0.1% Triton X-100 and 1% bovine serum albumin (BSA, Sigma‒Aldrich) in PBS for 2 h at RT. The samples were then incubated for three days at + 4 °C with the following primary antibodies diluted in PBS with 1% NDS, 1% BSA, and 0.1% Triton X-100: monoclonal anti-β-tubulin III (βtub_III_, chicken, 1:100, ab41489, Abcam), anti-βtub_III_ (rabbit, 1:250, ab52623, Abcam) anti-microtubule-associated protein 2 (MAP-2, rabbit, 1:200, AB5622, Millipore), anti-MAP-2 (chicken, 1:200, NB300-213, Novus), anti- cluster of differentiation 31 (CD31, mouse, 1:200, M0823, Dako), anti-CD31 (rabbit, 1:25, ab28364, Abcam), and anti-alpha smooth muscle actin (α-SMA, mouse, 1:300, ab7817, Abcam) antibodies. The samples were then washed four times with 1% BSA in DPBS. Thereafter, the samples were incubated with the following Alexa Fluor-labeled secondary antibodies diluted in DPBS with 1% BSA: goat anti-chicken 488 (A11039, 1:200), donkey anti-rabbit 568 (A10042, 1:200) and donkey anti-mouse (A31571, 1:125) (all from Thermo Fisher) overnight at + 4 °C followed by two washes with PBS. The samples were then washed with 1.0 µg/ml DAPI (Sigma‒Aldrich) in PBS for 1 h at RT, followed by daily washes for four days with PBS at + 4 °C. Confocal imaging was performed by using an LSM 780 laser scanning confocal microscope with a Quasar spectral GaAsP detector (all from Carl Zeiss). For fluorescence imaging, a DMi8 inverted microscope (Leica) was used. Images were processed with Fiji [39], Huygens Essentials (Scientific Volume Imaging) and Imaris (Oxford instruments) softwares.

### Calcium imaging

For calcium imaging experiments, 32 days differentiated hiPSC neurons were preplated on 6-well plates for 14 days before plating to multicultures according to the normal differentiation protocol [36]. Prematuration was performed to enhance the maturation of the cells for functional measurements before seeding the cells onto microfluidic chips. After the prematuration period, hiPSC neurons were plated to multicultures on day 46 as previously described (Fig. 1).

For calcium imaging, the cells were washed with extracellular solution (ECS, 137 mM NaCl, 20 mM HEPES, 5 mM KCl, 5 mM D-Glucose, 4.2 mM NaHCO_3_, 2 mM CaCl_2_, 1.2 mM MgCl_2_, 1 mM Na-pyruvate, and 0.44 mM KH_2_PO_4_) three times. Then, Fluo8-AM (5 μM, AAT Bioquest) or Fluo4-AM (4 μM, ab241082, Abcam) in ECS was added and incubated at + 37 °C for 1 h. After Fluo8-AM/Fluo4-AM loading, the cells were washed twice with ECS, first with a quick wash followed by a 30 min wash at + 37 °C. Time-lapse video recordings were acquired for 2–5 min using a fluorescence microscope (IX70, Olympus or Dmi8, Leica). Calcium oscillations were quantified with Fiji software [39]. Time-lapse images captured by fluorescence microscopy were converted to grayscale (16 bit) and regions of interest (ROIs) were analyzed on the basis of change in fluorescent intensity of the calcium indicator in circular regions of the somas. Calcium oscillations are represented by changes in average fluorescence intensity inside the defined ROIs. Graphs of the intensity changes were generated based on the ROI values.

### Perfusion and hydrogel integrity assay

To assess the integrity of the hydrogels and the perfusion capability of the formed vasculature, fluorescent beads were introduced to the microfluidic chip as described previously [16]. Briefly, 2 µm yellow‒green fluorescent microbeads (FluoSpheres Carboxylate-Modified Microspheres, 2% solids, Thermo Fisher Scientific) were introduced to the microfluidic chips containing the multicultures on Day 8 of culture and time-sequential images were captured. Gravity-based perfusion was generated between the two medium channels which resulted in an interstitial flow through the hydrogel. The images were acquired sequentially every 10 s for 2 min using an EVOS FL cell imaging system (Thermo Fisher Scientific) immediately after introducing the beads to the microfluidic chip.

### Protein secretion analysis

A Proteome Profiler Antibody Array Kit for human angiogenesis related proteins (Cat. ARY007, R&D Systems) was used to identify protein secretions. The manufacturer’s instructions were followed to investigate the relative levels of angiogenesis-related proteins from 4 different samples in EGM-2 including both BMSC and ASC multi- and cocultures. Images of the membranes were acquired using a ChemiDoc system with Image Lab software (Bio–Rad) and the spot pixel density was quantified using HLImage +  + Quickspots software (Western Vision).

### Vascular image analysis and statistical analysis

Image analysis was performed using Angiotool software [40] to calculate vessel area and total vessel length. Statistical analysis was performed using GraphPad Prism 9.0.0 (GraphPad Software, www.graphpad.com). Differences between groups were analyzed with nonparametric Mann–Whitney U test and differences were considered statistically significant at a *p* value < 0.05. (* Denotes *p* < 0.05.)

### Data availability statement

The data sets generated and analyzed for the current study are available from the corresponding author on reasonable request.

## Results

### Angiogenic medium supports the formation of 3D neurovascular networks on a chip

**Fig. 2:**
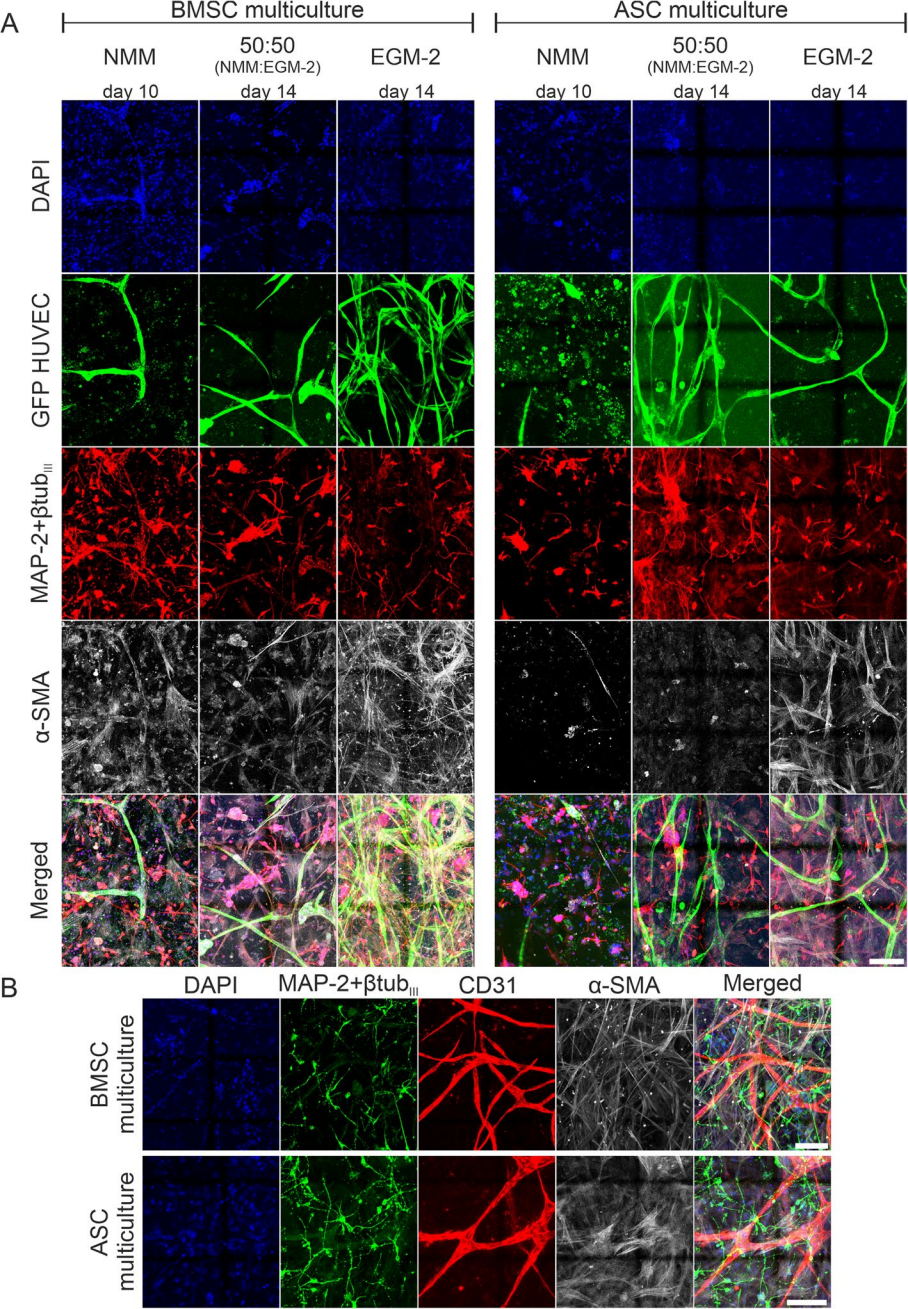
Medium optimization to support all the cell types in the multicultures. ICC staining of the BMSC and ASC multicultures on the microfluidic chip in different cell culture media on Day 10 (NMM) or Day 14 (50:50 (NMM:EGM-2) and EGM-2). **a** The GFP tag in the HUVECs (GFP HUVEC, green) showed that the formation of the vascular structures in both multiculture formats was best in EGM-2. The staining showed neuronal morphology (MAP-2 + βtubIII, red) in all the tested media. Pericytic properties of BMSCs and ASCs (α-SMA, gray) were greatest in EGM-2. **b** The staining for vascular differentiation marker (CD31, red) showed evidence of vasculature formation in EGM-2 in both multiculture formats on day 14 of culturing. Scale bar is 100 µm

The formation of neuronal and vascular networks in different culture media was investigated during the culturing period of 10 to 14 days to determine the conditions suitable for all cell types and the simultaneous formation of neuronal and vascular networks. Vasculature formation was successful in the angiogenic EGM-2 in the multicultures with mural cells of either BMSCs or ASCs origin (Fig. 2a). Vasculature formation in the multicultures in EGM-2 was confirmed by CD31 staining, which revealed the formation of vessel structures (Fig. 2b). In contrast, in NMM medium, negligible or no vasculature formation in multicultures was obtained with either mural cell type, as determined by the GFP signal of the HUVECs. In the 50:50 mixture medium (NMM:EGM-2), vasculature formation was more prominent than that in NMM but not as good as that in EGM-2 (Fig. 2a). Vasculature formation results in different media were further confirmed by quantifying the vessel area percentage (Supplementary Fig. 2). Based on the staining with neuronal markers MAP-2 and βtub_III_, neuronal morphology was less prone to medium influence than the vasculature as it was rather similar in all tested media in multicultures (Fig. 2a-b). BMSCs and ASCs showed the strongest pericytic properties in multicultures in EGM-2 indicated by the α-SMA staining (Fig. 2a-b). However, in NMM and 50:50 media, α-SMA-positive mural cells were not closely aligning with the vasculature. Especially with ASCs the mural cells seemed to die off in NMM and 50:50 media. Perfusion and hydrogel integrity assays indicated that the 3D samples remained mostly intact in EGM-2, in which the cells simultaneously formed perfusable vessel structures (Supplementary Fig. 3). Therefore, EGM-2 was selected as the culture medium for the 3D neurovascular networks on a chip based on its ability to support the growth and maturation of all the cell types in the multiculture for long periods.

### Neurons acquire functionality in 3D neurovascular networks on chip

**Fig. 3:**
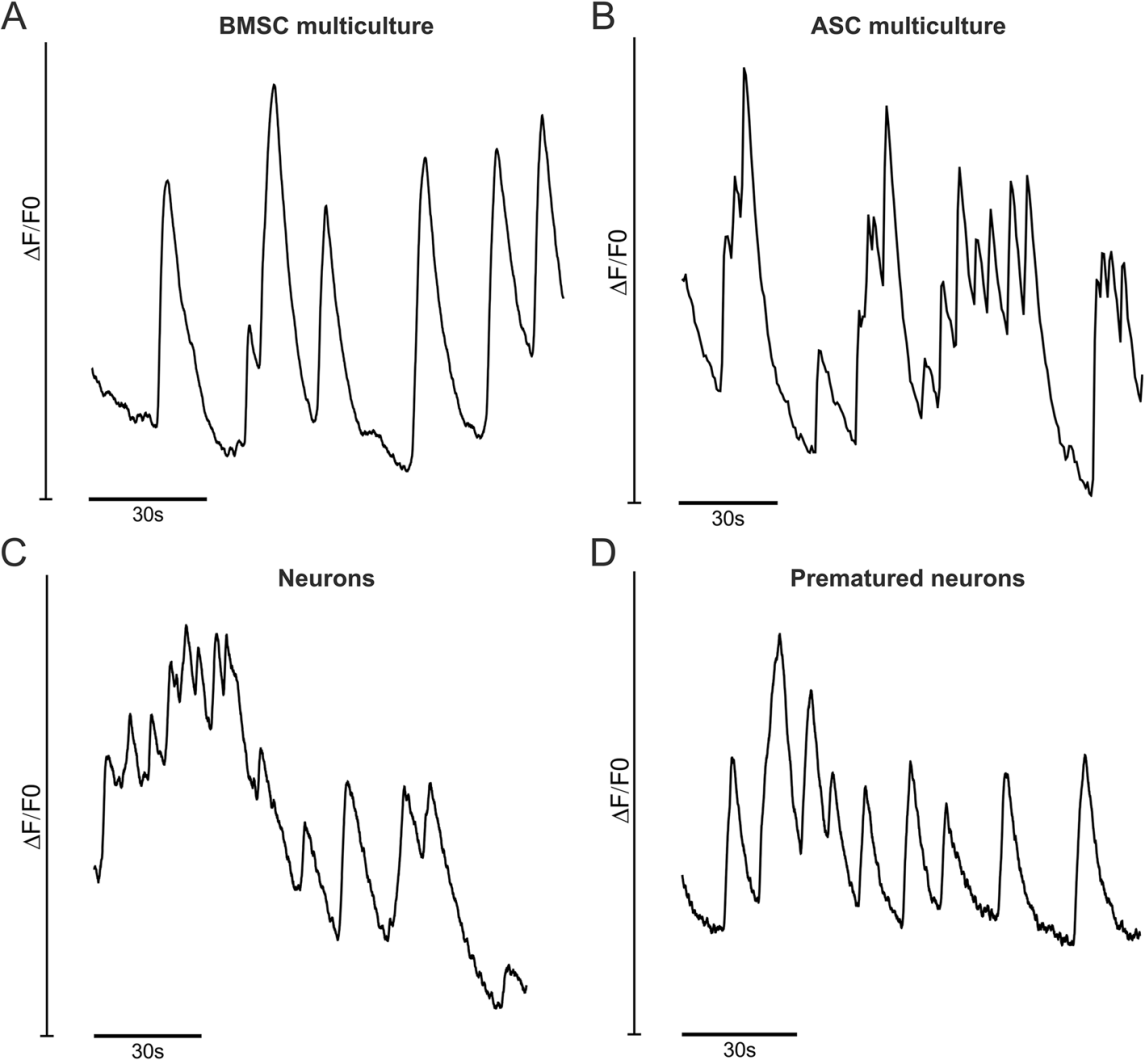
Calcium oscillations indicate neuronal functionality. Calcium imaging plots of 3D cultures on microfluidic chips showing the change in fluorescence intensity over the background (dF/F0) with respect to time. Calcium related neuron-specific activity was characterized by fast onset and slow decay. **a** Calcium transient activity of the BMSC multiculture plotted with respect to time to 120 s. **b** Calcium transient activity of the ASC multiculture plotted with respect to time to 180 s. **c** Calcium transient activity of hiPSC-derived neurons plotted with respect to time to 120 s. **d** Calcium transient activity of prematured hiPSC-derived neurons plotted with respect to time to 120 s

Angiogenic EGM-2 is optimized for culturing of ECs, as stated by the manufacturer, not for neuronal cells. As EGM-2 was selected as the culture medium for the 3D neurovascular networks on a chip, we also evaluated the functional maturation of the neurons in the multiculture. Neuronal activity was measured by calcium imaging (Fig. 3) for which the neurons were prematured for 14 days before seeding to multicultures (Fig. 1). Calcium oscillations were detected in neurons in the multicultures containing both mural cell types, i.e., BMSCs (Fig. 3a) and ASCs (Fig. 3b). Calcium imaging revealed functional neuronal connections with vascular structures (Supplementary Video 1). Functional neurons were also found in 3D neuronal monocultures including non-prematured (Fig. 3c) and prematured (Fig. 3d) cells cultured in NMM. Interestingly, the 3D neuronal monocultures cultured in EGM-2 did not express calcium oscillations (data not applicable) and depict unwanted behavior of cells by growing into medium channels and forming aggregates in hydrogel (Supplementary Fig. 4). These findings suggest that functional maturation of neurons was supported by the HUVECs and mural cells in the 3D neurovascular networks on a chip.

### Successful long-term culture of 3D neurovascular networks on a chip

**Fig. 4:**
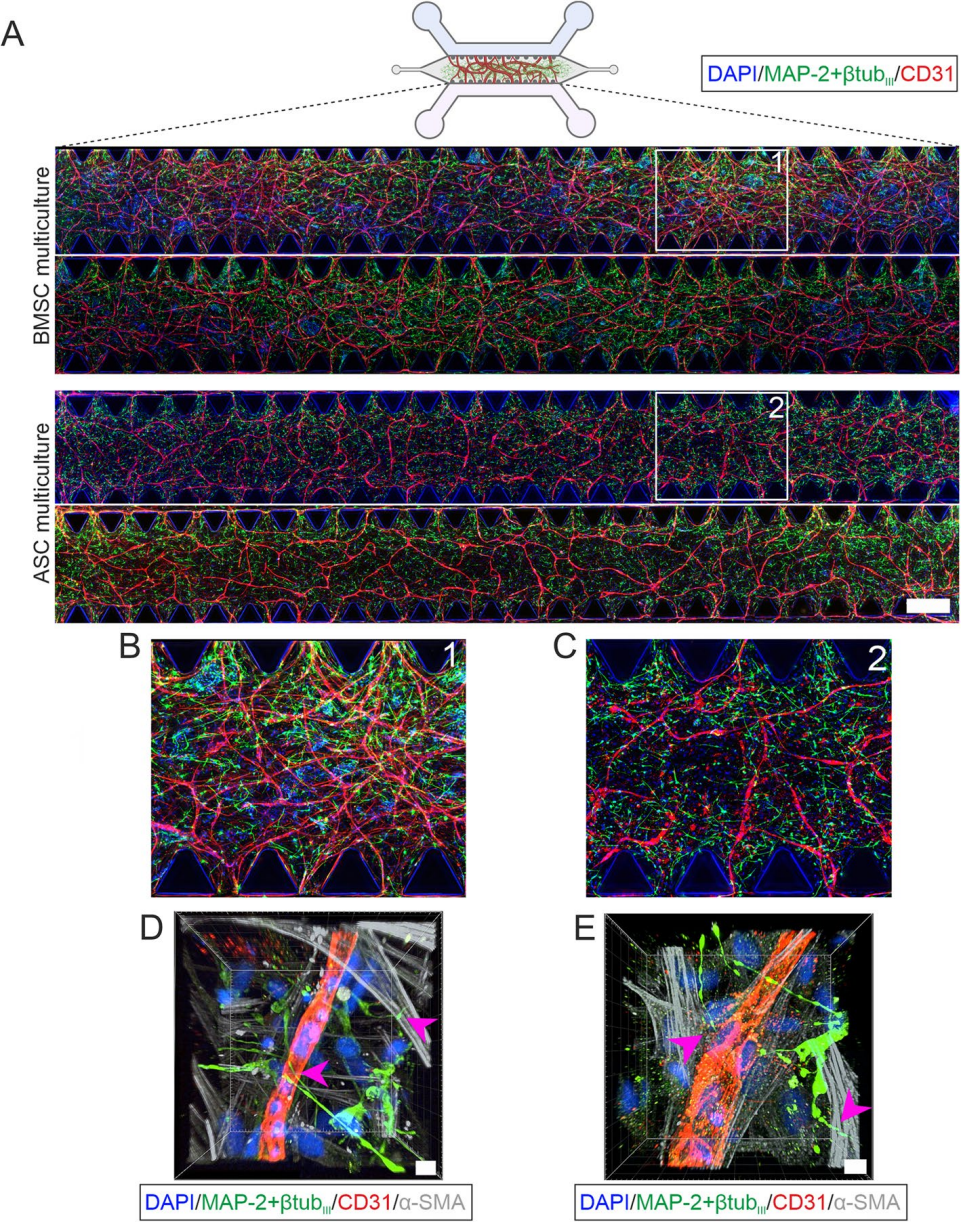
ICC staining of neurovascular networks on chip at day 14 timepoint. **a** Tilescan images showing the BMSC and ASC multicultures. Staining for a vascular differentiation marker (CD31, red) showed consistent and repeatable vasculature formation throughout the microfluidic chip in the BMSC and ASC multicultures. Staining for neuronal markers (MAP-2 + βtubIII, green) showed that a neuronal network had also formed throughout the hydrogel in both multiculture formats. Scale bar is 500 µm. **b** Close-up image of the BMSC multiculture. **c** Close-up image of the ASC multiculture. **d**-**e** Confocal 3D rendering of the multicultures showing neurons (MAP-2 + βtubIII, green) in the connections to both HUVECs (CD31, red) and mural cells (α-SMA, gray) in both the d BMSC and e ASC multicultures. Pink arrowheads indicate the neuronal connections to ECs and mural cells. Scale bar is 10 µm. The illustrations were created using Biorender.com

Immunocytochemical staining was performed to show the formation of neuronal and vascular networks and the differentiation of mural cells. Seeding hiPSC-derived cortical neurons, HUVECs and either BMSCs or ASCs together in collagen 1–fibrin hydrogel on a microfluidic chip resulted in the formation of 3D integrated neuronal and vascular networks that were maintained for at least 14 days in EGM-2 (Fig. 4a). The vascular network consistently formed throughout the hydrogel area on the microfluidic chip (Fig. 4a). Mural cells of both origins aligned with the formed vessels (Fig. 4d-e), supported the formation of the vasculature, and spread to all regions of the hydrogel (Supplementary Fig. 5c). Importantly, the neuronal network was evenly distributed among the vascular structures (Supplementary Fig. 5b). Integrated neuronal and vascular networks formed stable multicultures during the 14-day culture period, and they were reproducibly established; that is, the experiment was successfully repeated 7 times with BMSCs and 5 times with ASCs (Supplementary Table 3, Supplementary Fig. 6). Higher magnifications (Fig. 4b-c) and confocal imaging revealed that neurons formed neurite-mediated connections to vascular structures and mural cells throughout the hydrogel area (Supplementary Videos 2&3). Supplementing medium with the serine protease inhibitor aprotinin promoted the integrity of the hydrogel matrix by slowing the protease-mediated degradation of fibrin. Altogether, reproducible and stable 3D neurovascular networks with existing cellular connections were formed with mural cells of either origin.

### The neuronal network supports angiogenesis in 3D neurovascular networks on a chip

**Fig. 5:**
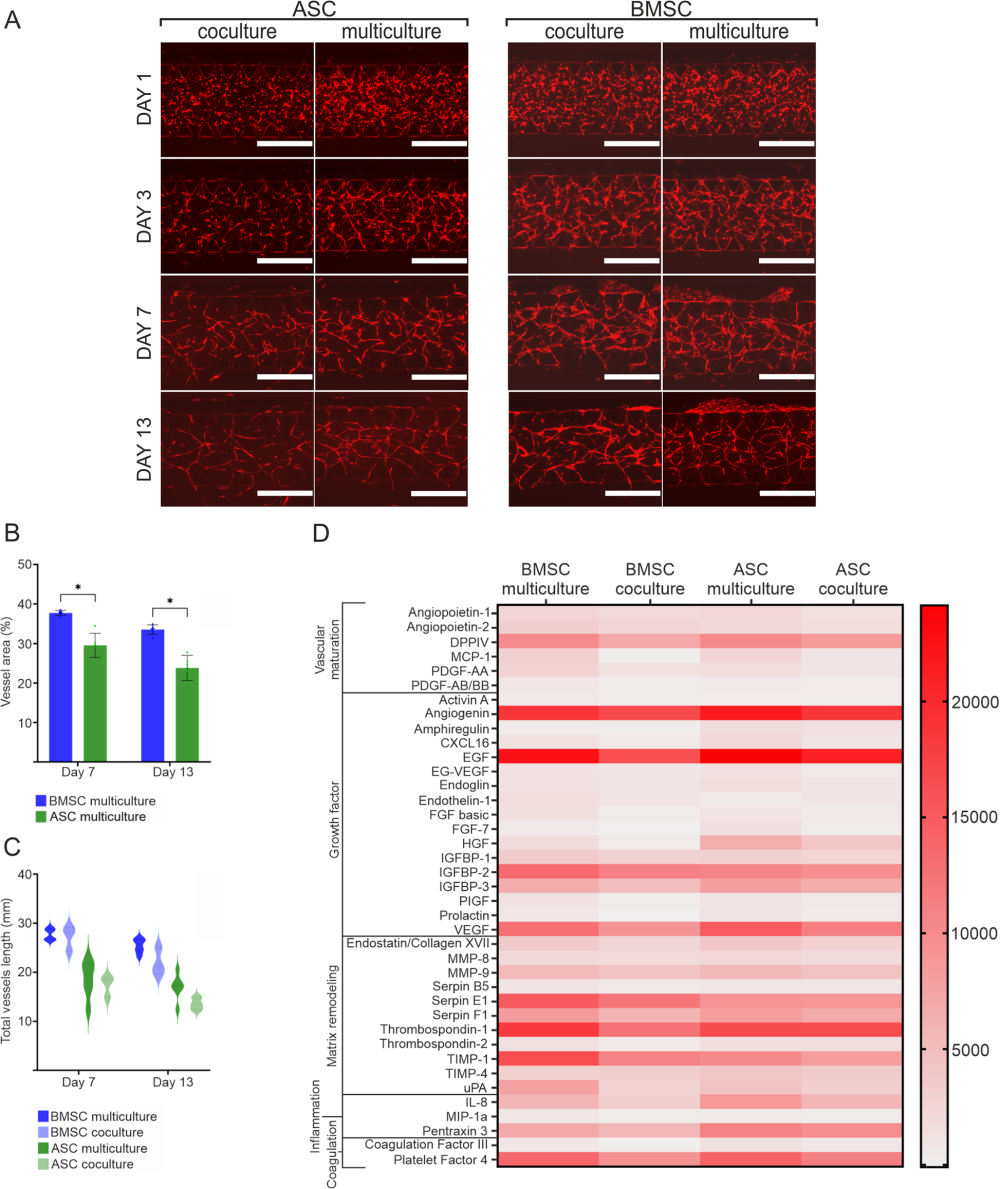
Differences in BMSC and ASC multi- and cocultures in vasculature formation and angiogenesis protein secretion. **a** Live image timelapse of vasculature formation in multi- and cocultures in EGM-2 indicated by the RFP tag of the HUVECs (RFP HUVEC, red) on Days 1, 3, 7 and 13 of the 14-day culture period. Scale bar is 1000 µm. **b** Angiotool was used to analyze the vessel area in the BMSC and ASC multicultures. **c** Angiotool was used to analyze the total vessel length in the BMSC and ASC multi- and cocultures in EGM-2. **d** Heatmap showing the Proteome Profiler angiogenesis protein secretion analysis of BMSC and ASC multi- and cocultures in EGM-2 from day 13–14 pooled samples

To study the effect of neurons in the 3D neurovascular networks, BMSC and ASC multi- and cocultures were compared. Multicultures consisted of neurons, ECs, and mural cells, while cocultures consisted of ECs and mural cells. The effects of neurons on vasculature formation (Fig. 5a) and angiogenesis-related protein secretion (Fig. 5d) were investigated. Overall, vasculature formation was successful in both the multi- and cocultures independent of mural cell type. Plotting the total vessel length in multi- and cocultures with either mural cell type indicated that the vasculature formation was enhanced in the multicultures compared to cocultures, particularly at later timepoints (Fig. 5c, Supplementary Fig. 7). According to a vasculature area analysis, the BMSC multiculture contained denser vasculature than the ASC multiculture (Fig. 5b, *p* < 0.05).

To further determine neurovasculature interplay during network coformation and comaturation, a commercially available semiquantitative human angiogenesis array was used to detect the secretion profile of 55 angiogenesis-related proteins (Supplementary Table 4.) in medium collected from the multi- and cocultures. With the array, 39 different proteins were identified (Fig. 5d) based on spot intensity quantification (Supplementary Fig. 8b). A majority of the detected proteins, 38 with BMSC and 37 with ASC multicultures, were overexpressed in the multiculture medium when normalized to coculture medium (Supplementary Fig. 8a). These findings indicate that neurons exerted a strong positive net effect on vascularization independent of the mural cell types used. The identified proteins were associated with vascular maturation, growth factors, matrix remodeling, inflammation, and coagulation processes. For example, the levels of angiogenin, VEGF and IL-8 were similarly increased in multicultures with BMSCs or ASCs. Overall, the normalized expression levels were typically higher in the BMSC than in the ASC multiculture; for example, the levels of thrombospondin-1 and -2 and urokinase-type plasminogen activator (uPA) were higher in the BMSC than in the ASC multicultures. Taken together, the data suggested that neurons support physical vasculature formation by boosting angiogenesis.

## Discussion

Both neuronal and vascular networks are crucial for normal development, function, and homeostasis in most tissues in a co-orchestrated manner. To date, neurovascular interactions have been studied in vitro mainly in relation to model the BBB functions [32, 41–44]. Little work has addressed the cellular and molecular interactions of neuronal and vascular networks. To our knowledge, this is the first study to report the generation of a functional long-term model with simultaneous induction of neuronal and vascular networks. Here, we used a commercially available microfluidic chip platform for human cell-based multicultures, neurons, ECs, and mural cells, in collagen 1–fibrin matrices to establish reproducible 3D neurovascular networks on a chip.

Our results show that angiogenic EGM-2 supplemented with aprotinin supports the multiculture of neurons with ECs and mural cells in collagen 1–fibrin matrix for at least 14 days. The 3D hydrogel matrix was selected based on our previous works of utilizing fibrin matrix for 3D vasculature formation [16] and collagen 1 matrix for 3D neuronal network formation [26, 27]. The collagen 1–fibrin matrix remained intact in the EGM-2, whereas it shrank in the NMM and the 50:50 medium. Supplementing EGM-2 with the serine-protease inhibitor aprotinin slowed the rate of matrix degradation, thus promoting hydrogel integrity in the 3D cultures. Therefore, aprotinin is an advisable supplement for long-term neuronal cultures in a fibrin-containing matrix, as fast degradation rates of fibrin scaffolds by the neurons have been previously reported [45, 46].

In multicultures, EGM-2 supported more extensive formation of vascular structures as well as greater pericytic properties of mural cells than either the NMM or 50:50 medium. EGM-2 includes e.g., FGF-B, VEGF, recombinant analog of insulin-like growth factor (R3-IGF) and human epidermal growth factor (hEGF) and has been used successfully to form in vitro vascularization with HUVECs and BMSCs or ACSs as mural cells in a fibrin matrix [16]. It has also been used in direct cocultures of HUVECs with chicken or rat dorsal root ganglia (DRGs) neuronal explants, and it produced enhanced axonal growth compared to that obtained with DMEM [47]. In contrast to the clear positive effect of EGM-2 on vasculature formation, neuronal network formation was not robustly affected by the medium choice. Many supplements in EGM-2, such as FGF-B, IGF, and EGF are also commonly used in neuronal culture media [36, 48, 49] which explains why neuronal differentiation was not affected by the medium choice. Overall, aprotinin-supplemented EGM-2 together with a collagen 1–fibrin matrix provided a stable environment for the neurovascular networks on the chip model.

The vasculature formation and function in multicultures were verified by imaging GFP- or RFP-HUVECs, staining for CD31 and α-SMA, and particle perfusion analysis. Neuronal networks were stained for MAP-2 and βtub_III_ and functionality was evaluated with calcium imaging. The staining revealed the consistent formation of both neuronal and vascular networks throughout the hydrogel area, which was repeatable between experiments. The neuronal networks formed very similarly in the multicultures independent of the mural cell types used, which had not been addressed in previous works. In line with a previous study [30], our study verified that neurons were functional in multiculture, and the calcium oscillation patterns were similar to the patterns presented in previous study [30]. Functional neurons were also detected in control 3D monocultures in NMM; however, none were detected in 3D monocultures cultured in EGM-2. Thus, EGM-2 does not seem to be sufficient to support the functional maturation of neurons, and other cell types in the multicultures are required for it. In line with this, ECs have been shown to promote the maturation and functionality of mouse cortical neurons in either 2D direct cocultures or in EC-conditioned medium [50]. The formed vasculature enabled particle flow independent of the mural cell type utilized. The vasculature formation was, however, more robust and more precisely aligned in the BMSC multicultures than in the ASC multicultures, which was consistent with our previous findings [16]. Obviously, the donor source can also affect the vascularization properties [16]. However, it would require more extensive donor comparison to draw conclusions on this matter. Interestingly, when comparing the multicultures with cocultures, the multicultures with the mural cells of either origin seemed to show either similar or slightly more extensive vasculature than the respective cocultures. These results imply an angiogenesis-inducing role of the neurons in the multicultures. Previously, human embryonic stem cell (hESC) -derived sensory neurons had been found to promote angiogenesis in both Transwell and direct coculture approaches [20]. Taken together, our results support the contact-mediated promotion of angiogenesis in multicultures and suggest that hiPSC-derived neurons have angiogenesis-inducing potential in a perfusable microphysiological 3D environment.

To further analyze the angiogenesis-inducing potential of neurons, we investigated the secretion of angiogenesis-related proteins in the multi- and cocultures. The analysis included proteins that are involved in vascular maturation, growth factors, matrix remodeling, inflammation, and coagulation processes. In the multicultures of ECs, mural cells and neurons, the secretion of majority (~ 70%) of the detected angiogenesis-related proteins was increased compared to that in cocultures with ECs and mural cells. Neurons can express and secrete angiogenic factors [51]. The degree of secretion increase in multicultures was rather surprising, as previously it had been shown that sole neuronal cultures exhibited limited potential to secrete angiogenic factors (~ 30%, 6 out of 20 proteins) [20]. The differences in protein secretion between multi- and cocultures may have been partly a result from neuronal secretion of angiogenic proteins [20], but the effect was more likely caused by neurovascular cell crosstalk, in which neurons induced secretion from the ECs or mural cells [47]. These possibilities do not rule out one another, and the outcome may involve synergy of both effects. The increased secretion profiles were almost identical in multicultures independent of the mural cell type; however, the relative secretion levels were higher in the BMSC-containing cultures than in the ASC-containing cultures. In line with a previous study, the expression of genes related to vasculature formation has been shown to be higher in cocultures comprising ECs and BMSCs than in cocultures comprising ECs and ACSs [16]. As both mural cell types successfully support the neurovascular network formation, future studies are warranted to evaluate whether their origin exerts an impact in disease-mimicking conditions, such as hypoxia.

In relation to vascular maturation and growth factors, elevated secretion of angiopoietin-1 (ANG-1), platelet-derived growth factor (PDGF)-AB/BB and VEGF was detected in the multicultures. ANG-1 and PDGF have been shown to participate in the interaction between ECs and mural cells [52], with PDGF considered to be a mediator of signaling from ECs to mural cells; ANG-1 shows the reverse signaling direction [53]. In this study, this increased signaling was most likely a result of neurovascular crosstalk, not direct neuronal secretion. VEGF is one of the most essential growth factors participating the crosstalk between neuronal and vascular networks [54]. It is produced mainly by other cell types than ECs, such as mural cells and neurons [55, 56]. In the multicultures the secretion level of VEGF was greatly increased suggesting its meaningful role in vascular and neuronal patterning during development [57]. It has been shown that sensory neurons can modulate the transcriptional and translational profiles of ECs to promote extracellular matrix (ECM) remodeling, involving the activities of matrix metalloproteinases (MMPs) [58]. In line with this, our findings showed an increase in the protein levels of serpins, thrombospondins, TIMPs, uPA, endostatin and MMPs in the multicultures. Some of these factors, such as uPA, are secreted by both neurons and ECs [59]. In vascular structures, uPA regulates angiogenesis and vascular permeability through the proteolytic degradation of the ECM [60]. It is also expressed in axonal growth cones and participates in the regulation of neuronal migration and axon growth [61]. Altogether, proteins that act in matrix remodeling are crucial to angiogenesis and vascular remodeling, as these invasive processes require cell migration and degradation of the basement membrane of ECs [62]. Thus, our findings support previous observations and emphasize the role of neurons in vascular remodeling in the proposed in vitro concept.

Regarding inflammation and coagulation associated proteins, the secretion of interleukin (IL)-8 and platelet factor 4 (PF4) was highly elevated in the multicultures. The secretion of IL-8, along with other proangiogenic factors such as GRO, TIMP-1, TIMP-2, EGF and MCP-1, by neurons has been shown in a recent study [20], and of these factors, TIMP-1 and EGF were also detected in our multicultures. IL-8 is an inflammatory cytokine that enhances EC survival and tube formation [63] and exerts neurotropic effects [64]. In multicultures, ECs are the most likely source for IL-8 secretion [65]. PF4 is a coagulation cascade related inflammatory chemokine that has been shown to affect angiogenesis, especially via mural cells, by inducing cell proliferation [66]. Altogether, these results indicate the pervasive role of neurovascular crosstalk in signaling pathways from the cellular to network level.

## Conclusions

The present study demonstrates the successful development of a 3D neurovascular OoC model with a commercially available microfluidic chip. The characteristics of the neuronal and vascular networks were morphologically and functionally validated. Of the mural cell types tested, BMSCs and ASCs, both supported neurovascular network formation; however, the BMSCs performed slightly better. The angiogenic process was substantially elevated in the multicultures, implying the importance of neurovascular crosstalk even in the in vitro setup. The formation of both neuronal and vascular networks is critical for physiologically relevant OoC and BoC modeling. The model enables further study and understanding of the interactions between neuronal and vascular networks and is applicable to the development of vascularized and innervated OoCs.

## Supplementary Information

**Additional file 1: Supplementary Videos.**

**Additional file 2:** **Supplementary Material.** **Supplementary Fig. 1.** Neurons in collagen I-fibrin hydrogel in different medium without aprotinin supplementation on day 3 of culturing. Degradation and shrinkage can be seen rapidly after plating the cells to hydrogel. Scale bar is 500 μm. **Supplementary Fig. 2.** Angiotool was used to analyze the vessel area percentage in the BMSC and ASC multicultures in NMM, 50:50 and EGM-2 media. **Supplementary Fig. 3.** Hydrogel integrity is affected by the cell culture media. a Hydrogel integrity test with fluorescent beads for multicultures in the microfluidic chip in different cell culture media at day 8 timepoint. Interstitial flow through hydrogel carries fluorescent beads across the hydrogel region. Beads flow more freely through hydrogel in NMM and 50:50 media, whereas in EGM-2 beads follow the vascular structures. White arrows indicate the flow of the beads. Scale bar is 1000 μm. b Phase-contrast images of multicultures in different cell culture media at day 9 timepoint. Images showed shrinkage of hydrogel in microfluidic chips in BMSC and ASC multicultures in NMM and 50:50 media. Scale bar is 500 μm. **Supplementary Fig. 4.** Neurons stained with neuronal markers βtubIII+MAP-2 in different medium in neuronal monocultures and in multicultures of neurons, ECs, and BMSCs/ASCs on day 14. a Tilescan images of neurons in microfluidic chips. In EGM-2 neuronal monoculture, neurons grow to medium channels and form aggregates in the hydrogel. In EGM-2 multicultures or NMM neuronal monoculture these effects cannot be seen, as only few cells grow in the channels compared to EGM-2 monocultures. Scale bar is 500 μm. b Close ups of the tilescan images of neurons in different media in EGM monoculture, NMM monoculture and multicultures with BMSCs or ASCs as the mural cell type. **Supplementary Fig. 5.** Formation of vascularization, neuronal networks and supporting mural cells in the multicultures. a Live image timelapse of the formation of vascular structures indicated by the GFP tag of HUVECs trough the 14-day culturing period in BMSC and ASC multicultures. Scale bar is 1000 μm. b ICC staining of multicultures with neuronal markers showed the similar formation of neuronal networks in both multiculture formats. c ICC staining of multicultures with mural cell marker showed the pericytic characteristics of BMSCs and ASCs to cells in multicultures at day 14 timepoint. Both BMSCs and ASCs expressed the mural cell marker and spread throughout the multicultures. However, the morphology of the differentiated mural cells was different when using cells of different origin. Scale bar is 500 μm. **Supplementary Fig. 6.** Reproducibly established vasculature in multicultures in different experiments. a Representative live images of the vasculature formation in BMSC and ASC multicultures indicated by the GFP or RFP tag of HUVECs on days 13-14 of culturing. Scale bar is 1000 μm. b Angiotool was used to analyze the vessel area in the BMSC and ASC multicultures in different experiments. BMSC and ASC cell lines used in experiments are color coded with different shades of blue and green. In experiments 4 and 5 ASCs were not utilized. More detailed information of the cell lines is listed in supplementary tables 1,2 and 3. **Supplementary Fig. 7.** Angiotool was used to analyze the vessel area percentage in the BMSC and ASC multi- and cocultures in EGM-2. **Supplementary Fig. 8.** Proteome Profiler human angiogenesis array analysis. a Protein secretion profile of BMSC and ASC multicultures normalized to BMSC and ASC cocultures, respectively. Most of the angiogenesis-related proteins are overexpressed in multicultures compared to cocultures. b Proteome Profiler membranes from BMSC and ASC multi- and cocultures used for spot intensity quantification. Medium for analysis was collected from experiment 6. Details of the BMSC and ASC cell lines used in the experiment are listed in Supplementary Tables 1, 2 and 3. **Supplementary Table 1.** Donor information for the used primary mesenchymal stem/stromal cell lines BMSC 1-2 and ASC 1-2. For donor cell line BMSC 1, Body Mass Indexwas not available. **Supplementary Table 2.** Surface marker expression of the studied donor BMSCs and ASCs. Individual donor cell line passage during analysis is denoted in the column ‘’P’’. Positive > 80%; low < 10%; negative < 2%. The cells were characterized as MSCs due to positive expression of CD73, CD90, and CD105, and low or negative expression of CD14, CD19, CD45 [67]. The expression of CD34 and HLA-DR was present at variable levels. The heterogeneity in surface marker expression if compared to the ISCT requirements could be explained by changes in cell culturing conditions [68]. **Supplementary Table 3.** Experimental information of the used primary mesenchymal stem/stromal cell lines BMSC 1-2 and ASC 1-2. Experiments were repeated 7 and 5 times for BMSCs and ASCs, respectively. **Supplementary Table 4.** Proteins analyzed with the Proteome Profiler array.

## Data Availability

The datasets used and/or analyzed during the current study are available from the corresponding author on reasonable request.

## Abbreviations

5050: 50:50 Mixture medium of EGM-2 and NMM
αMEM: α-Modified minimum essential medium
α-SMA: Alpha smooth muscle actin
βtub_III_: β-Tubulin III
AA: Ascorbic acid
ANG-1: Angiopoietin-1
ASC: Adipose tissue-derived stem/stromal cell
BBB: Blood–brain barrier
BDNF: Brain derived growth factor
BMSC: Bone-marrow-derived stem/stromal cell
BoC: Body-on-chip
BSA: Bovine serum albumin
CD31: Cluster of differentiation 31
CNS: Central nervous system
Coculture: Culture of ECs and mural cells (BMSC/ASC)
DRG: Dorsal root ganglia
db-cAMP: Dibutyryl-cyclic AMP
EBM-2: Endothelial Cell Growth Basal Medium
EC: Endothelial cell
ECM: Extra cellular matrix
EGF: Epidermal growth factor
EGM-2: Endothelial Cell Growth Medium-2
FGF-B: Basic fibroblast growth factor
GDNF: Glial derived growth factor
GFP: Green fluorescent protein
hESC: Human embryonic stem cell
hiPSC: Human induced pluripotent stem cell
HS: Human serum
HUVEC: Human umbilical vein endothelial cell
IL-8: Interleukin-8
MAP-2: Microtubule-associated protein 2
MMP: Matrix metalloproteinase
MS: Multiple sclerosis
MSC: Mesenchymal stem/stromal cell
Monoculture: Culture of neurons
Multiculture: Culture of neurons, ECs, and mural cells (BMSCs/ASCs)
NDS: Normal donkey serum
NMM: Neural maturation medium
OoC: Organ-on-chip
PDGF: Platelet-derived growth factor
PF4: Platelet factor 4
R3-IGF: Recombinant analog of insulin-like growth factor
RFP: Red fluorescent protein
RT: Room temperature
uPA: Urokinase-type plasminogen activator
VEGF: Vascular endothelial growth factor
