# Supplementary material for: Simultaneous induction of vasculature and neuronal network formation on a chip reveals a dynamic interrelationship between cell types
Source: Cell Commun Signal. 2023 Jun 14;21:132. doi: 10.1186/s12964-023-01159-4 (PMC10265920; doi:10.1186/s12964-023-01159-4)
Supplement: Supplementary file 2 — Additional file 1: Supplementary Videos. [file 12964_2023_1159_MOESM1_ESM.pptx]

## Slide 1
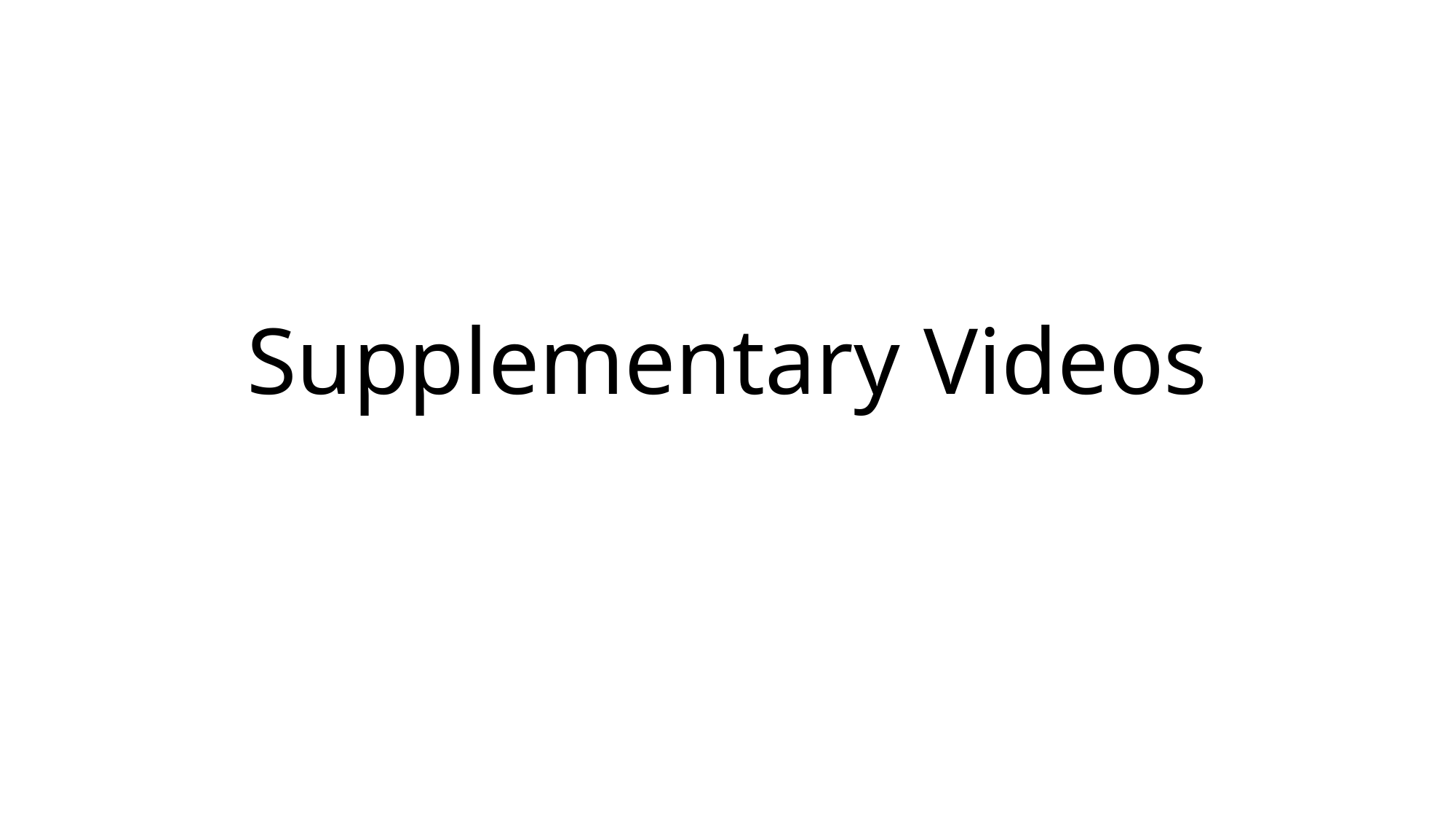

# Supplementary Videos

## Slide 2
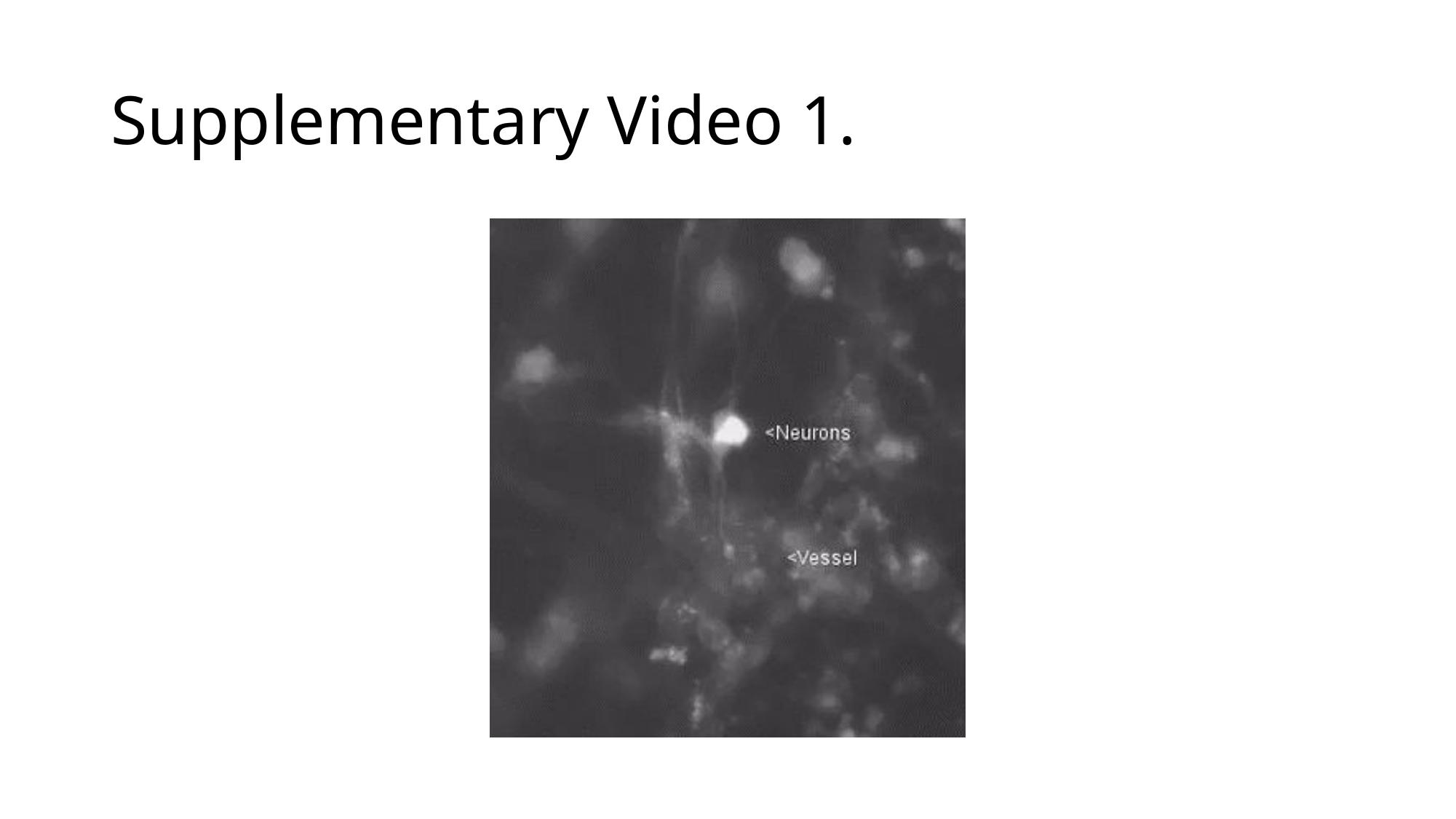

# Supplementary Video 1.

## Slide 3
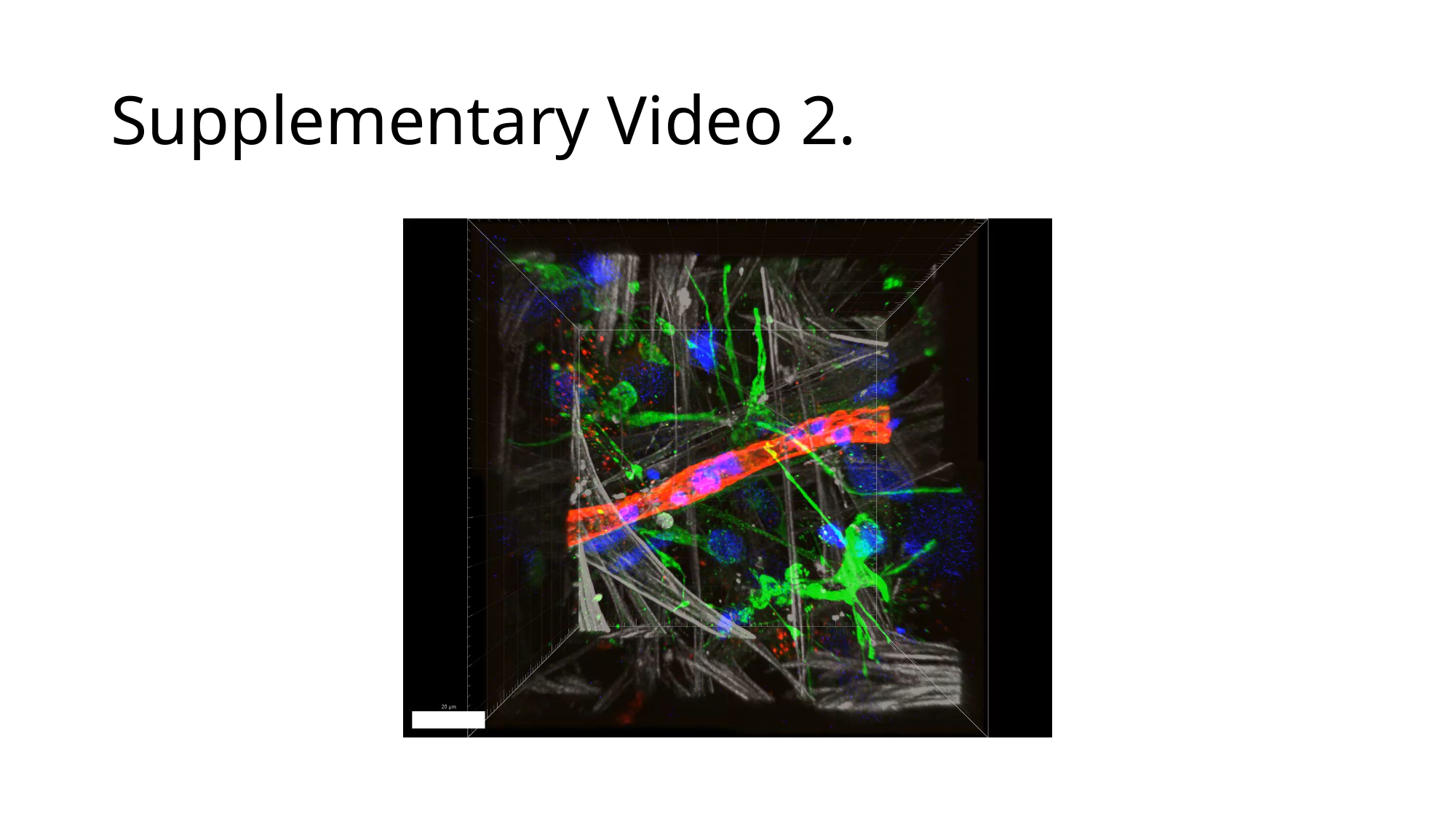

# Supplementary Video 2.

## Slide 4
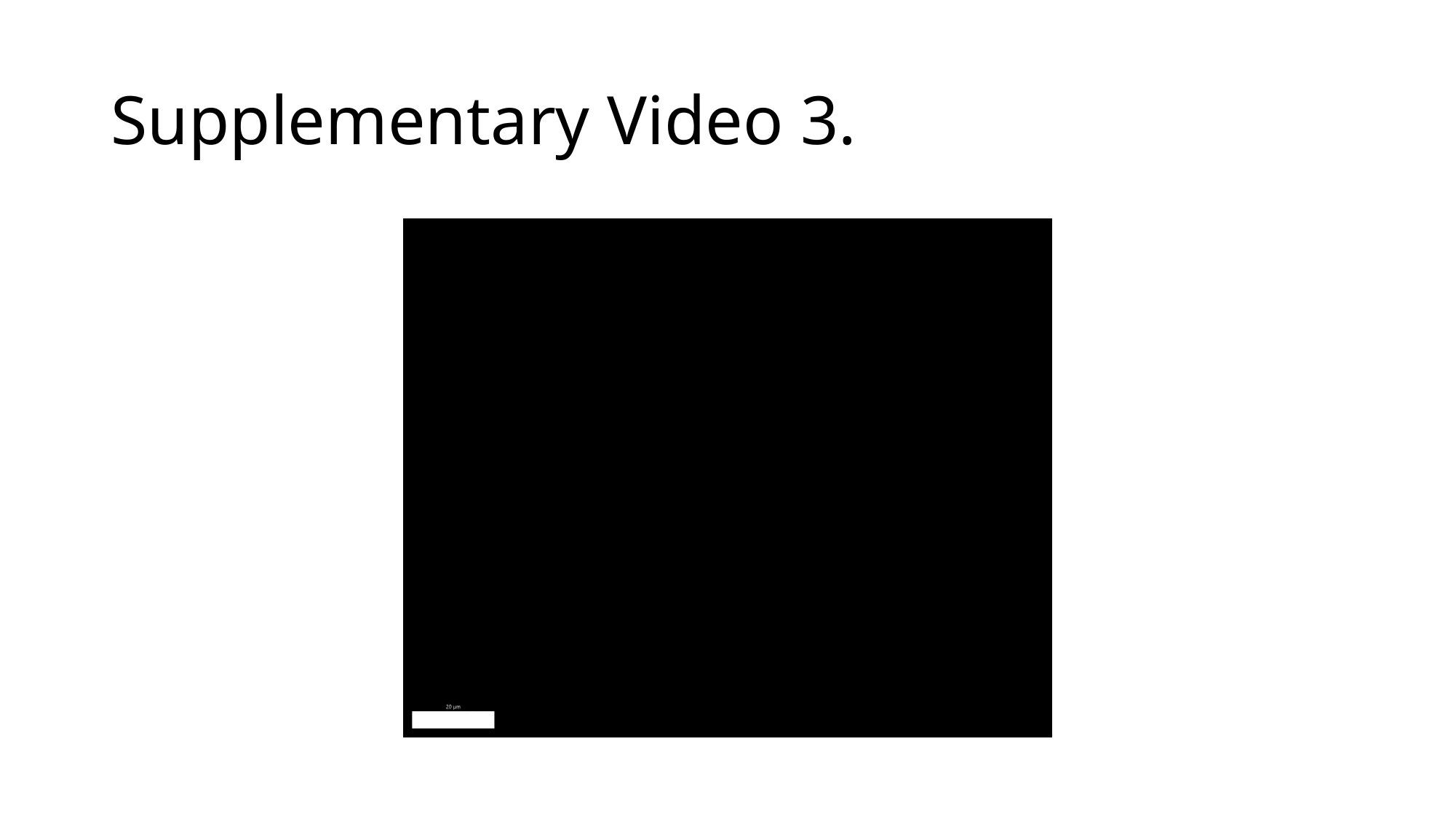

# Supplementary Video 3.
