# Supplementary material for: Simultaneous induction of vasculature and neuronal network formation on a chip reveals a dynamic interrelationship between cell types
Source: Cell Commun Signal. 2023 Jun 14;21:132. doi: 10.1186/s12964-023-01159-4 (PMC10265920; doi:10.1186/s12964-023-01159-4)
Supplement: Supplementary file 3 — Additional file 2: Supplementary Material. Supplementary Fig. 1. Neurons in collagen I-fibrin hydrogel in different medium without aprotinin supplementation on day 3 of culturing. Degradation and shrinkage can be seen rapidly after plating the cells to hydrogel. Scale bar is 500 μm. Supplementary Fig. 2. Angiotool was used to analyze the vessel area percentage in the BMSC and ASC multicultures in NMM, 50:50 and EGM-2 media. Supplementary Fig. 3. Hydrogel integrity is affected by the cell culture media. a Hydrogel integrity test with fluorescent beads for multicultures in the microfluidic chip in different cell culture media at day 8 timepoint. Interstitial flow through hydrogel carries fluorescent beads across the hydrogel region. Beads flow more freely through hydrogel in NMM and 50:50 media, whereas in EGM-2 beads follow the vascular structures. White arrows indicate the flow of the beads. Scale bar is 1000 μm. b Phase-contrast images of multicultures in different cell culture media at day 9 timepoint. Images showed shrinkage of hydrogel in microfluidic chips in BMSC and ASC multicultures in NMM and 50:50 media. Scale bar is 500 μm. Supplementary Fig. 4. Neurons stained with neuronal markers βtubIII+MAP-2 in different medium in neuronal monocultures and in multicultures of neurons, ECs, and BMSCs/ASCs on day 14. a Tilescan images of neurons in microfluidic chips. In EGM-2 neuronal monoculture, neurons grow to medium channels and form aggregates in the hydrogel. In EGM-2 multicultures or NMM neuronal monoculture these effects cannot be seen, as only few cells grow in the channels compared to EGM-2 monocultures. Scale bar is 500 μm. b Close ups of the tilescan images of neurons in different media in EGM monoculture, NMM monoculture and multicultures with BMSCs or ASCs as the mural cell type. Supplementary Fig. 5. Formation of vascularization, neuronal networks and supporting mural cells in the multicultures. a Live image timelapse of the formation of vascular struct [file 12964_2023_1159_MOESM2_ESM.pdf]

Supplementary Material

Supplementary Figures

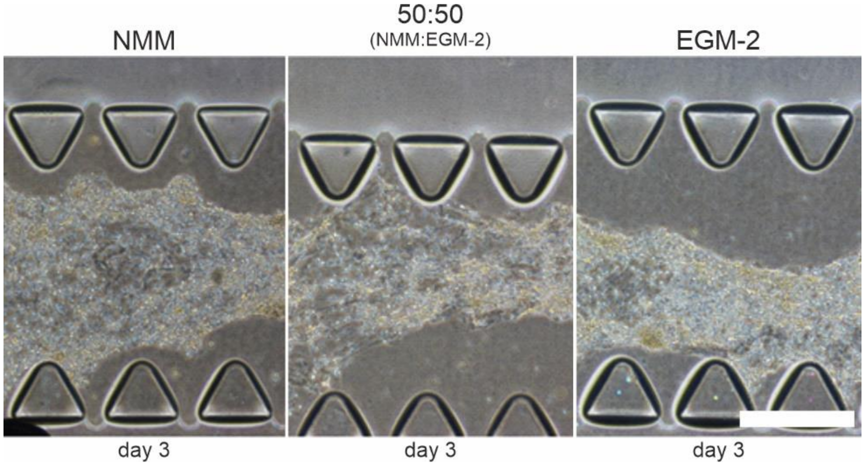

Supplementary Fig. 1 Neurons in collagen I-fibrin hydrogel in different medium without aprotinin supplementation on day 3 of culturing. Degradation and shrinkage can be seen rapidly after plating the cells to hydrogel. Scale bar is 500 µm.

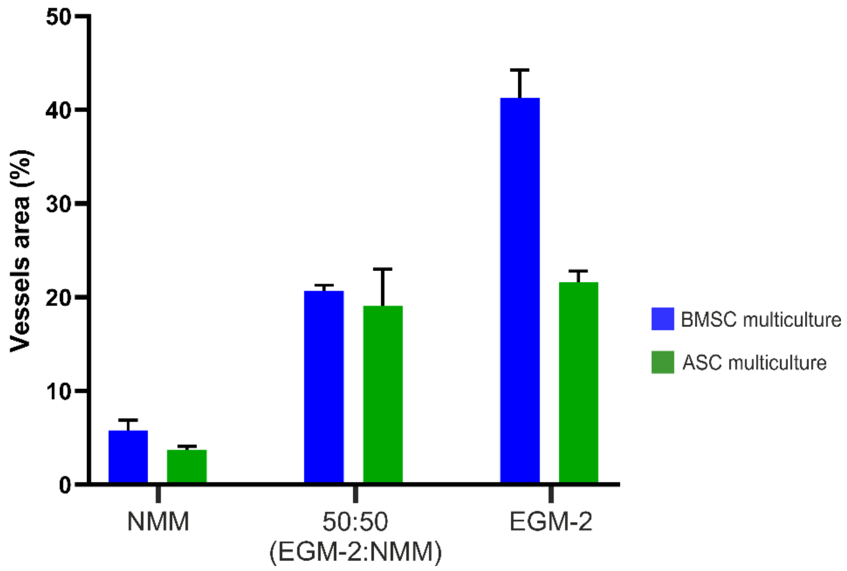

Supplementary Fig. 2 Angiotool was used to analyze the vessel area percentage in the BMSC and ASC multicultures in NMM (day 10), 50:50 (day 14) and EGM-2 (day 14) media.

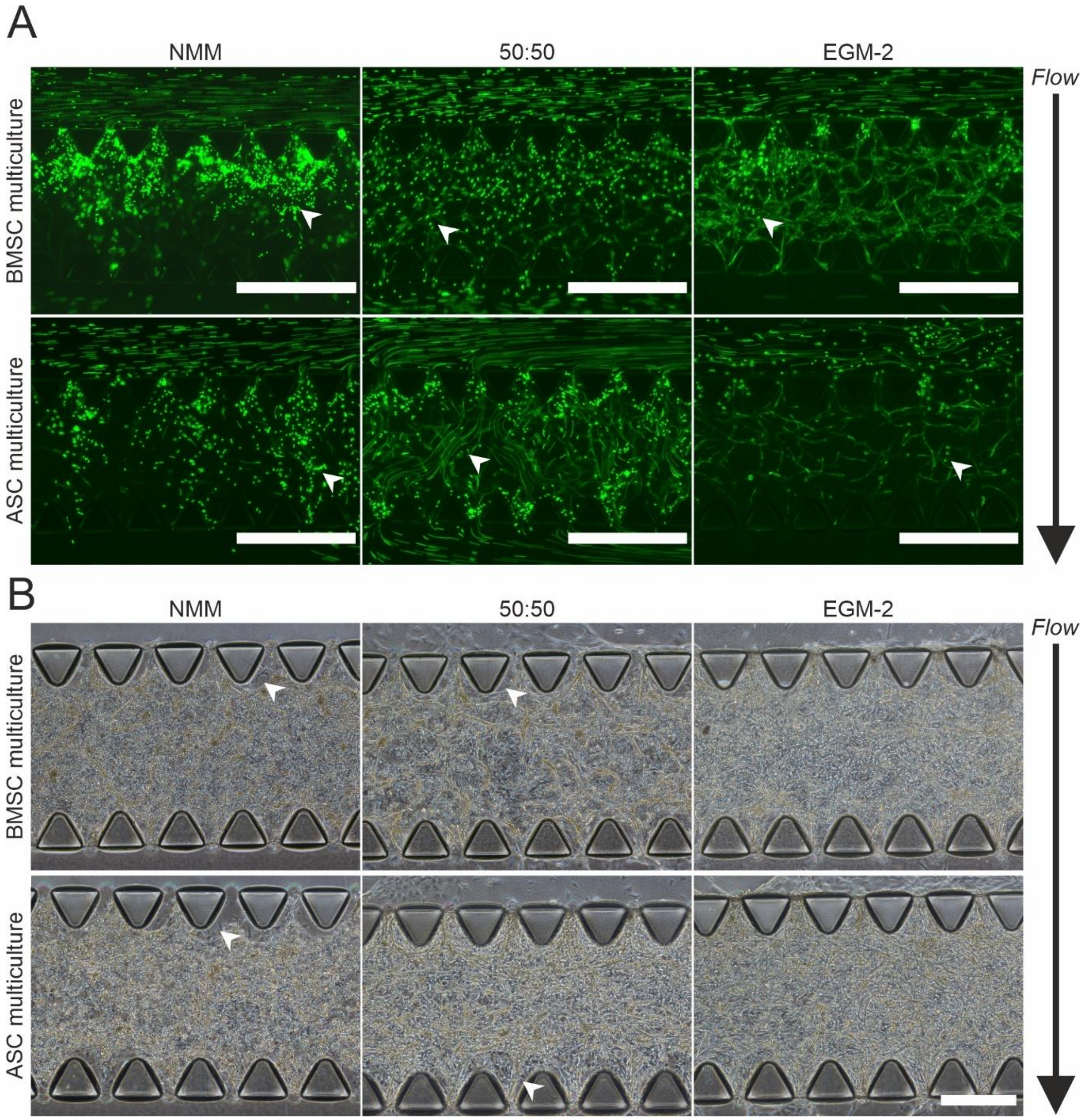

Supplementary Fig. 3 Hydrogel integrity is affected by the cell culture media. **a** Hydrogel integrity test with fluorescent beads for multicultures in the microfluidic chip in different cell culture media at day 8 timepoint. Interstitial flow through hydrogel carries fluorescent beads across the hydrogel region. Beads flow more freely through hydrogel in NMM and 50:50 media, whereas in EGM-2 beads follow the vascular structures. White arrows indicate the flow of the beads. Scale bar is 1000 µm. **b** Phase-contrast images of multicultures in different cell culture media at day 9 timepoint. Images showed shrinkage of hydrogel (white arrows) in microfluidic chips in BMSC and ASC multicultures in NMM and 50:50 media. Scale bar is 500 µm.

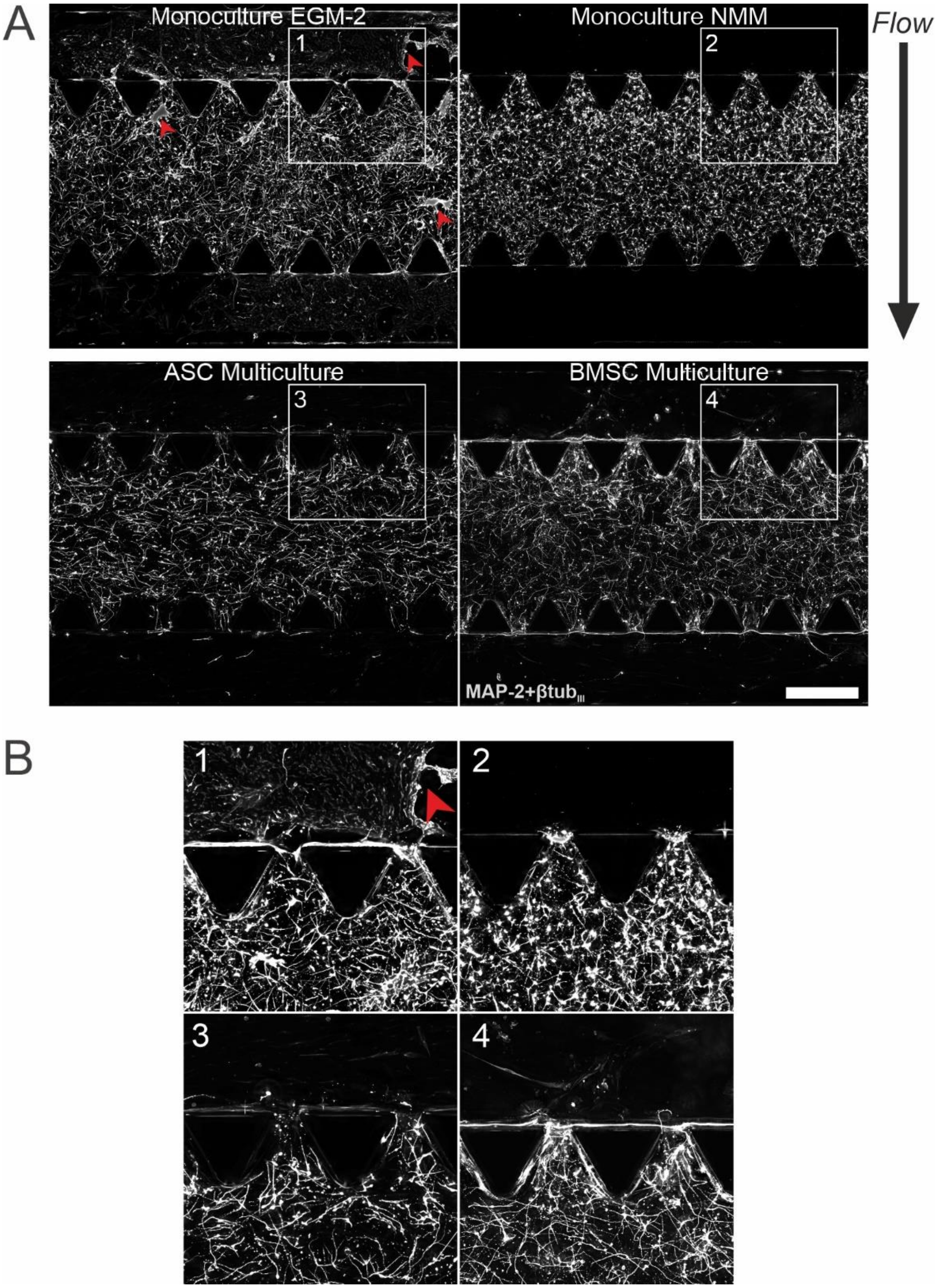

Supplementary Fig. 4 Neurons stained with neuronal markers βtub_{III}+MAP-2 in different medium in neuronal monocultures (EGM-2 and NMM) and in multicultures of neurons, ECs, and BMSCs/ASCs (EGM-2) on day 14. **a** Tilescan images of neurons in microfluidic chips. In EGM-2 neuronal monoculture, neurons grow to medium channels and form aggregates in the hydrogel (red arrowheads). In EGM-2 multicultures or NMM neuronal monoculture these effects cannot be seen, as only few cells grow in the channels compared to EGM-2 monocultures. Scale bar is 500 µm. **b** Close ups of the tilescan images of neurons in different media in EGM monoculture (1), NMM monoculture (2) and multicultures with BMSCs (3) or ASCs (4) as the mural cell type.

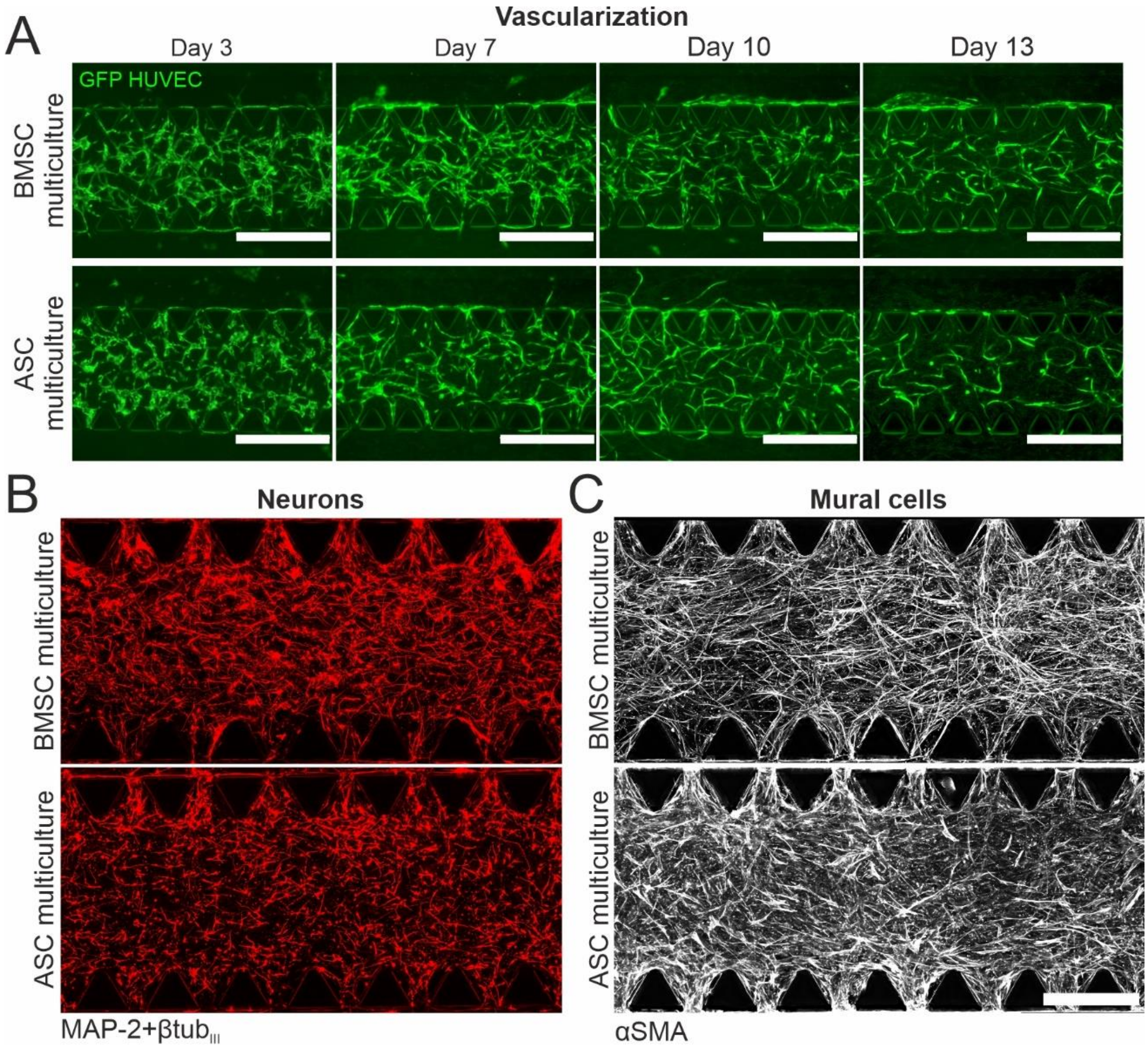

Supplementary Fig. 5 Formation of vascularization, neuronal networks and supporting mural cells in the multicultures. **a** Live image timelapse of the formation of vascular structures indicated by the GFP tag of HUVECs trough the 14-day culturing period in BMSC and ASC multicultures (GFP HUVEC, green). Scale bar is 1000 µm. **b** ICC staining of multicultures with neuronal markers (MAP-2+βtubIII, red) showed the similar formation of neuronal networks in both multiculture formats. **c** ICC staining of multicultures with mural cell marker (a-SMA, gray) showed the pericytic characteristics of BMSCs and ASCs to cells in multicultures at day 14 timepoint. Both BMSCs and ASCs expressed the mural cell marker and spread throughout the multicultures. However, the morphology of the differentiated mural cells was different when using cells of different origin. Scale bar is 500 µm.

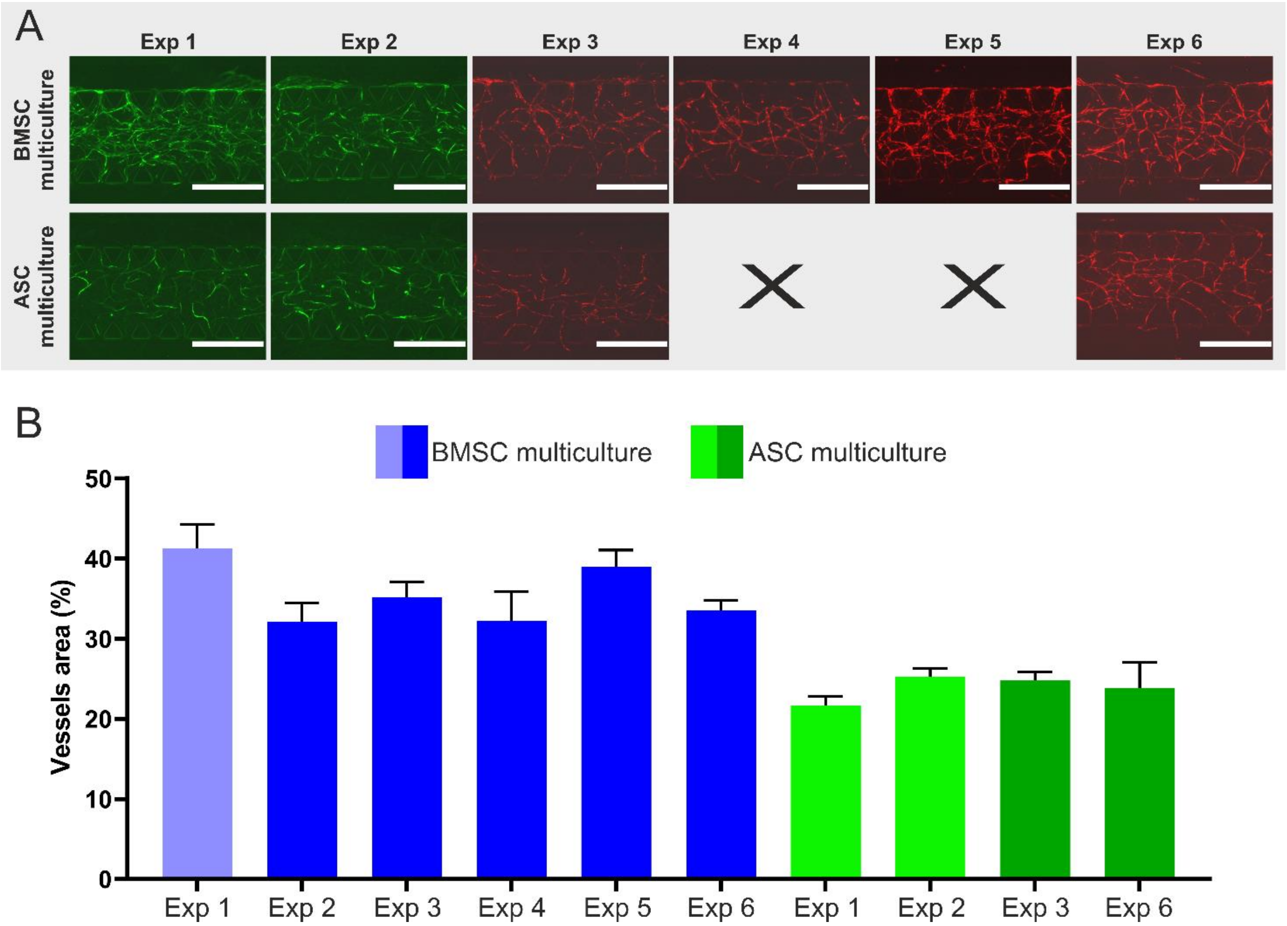

Supplementary Fig. 6 Reproducibly established vasculature in multicultures in different experiments (Exp 1-6). **a** Representative live images of the vasculature formation in BMSC and ASC multicultures indicated by the GFP or RFP tag of HUVECs on days 13-14 of culturing. Scale bar is 1000 µm. **b** Angiotool was used to analyze the vessel area in the BMSC and ASC multicultures in different experiments. BMSC and ASC cell lines used in experiments are color coded with different shades of blue (BMSC) and green (ASC). In experiments 4 and 5 ASCs were not utilized. More detailed information of the cell lines is listed in supplementary tables 1,2 and 3.

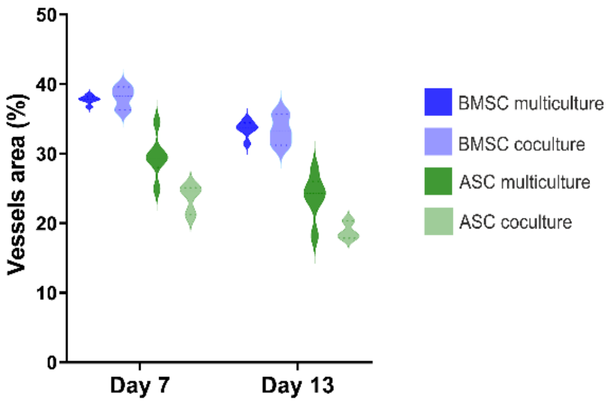

Supplementary Fig. 7 Angiotool was used to analyze the vessel area percentage in the BMSC and ASC multi- and cocultures in EGM-2.

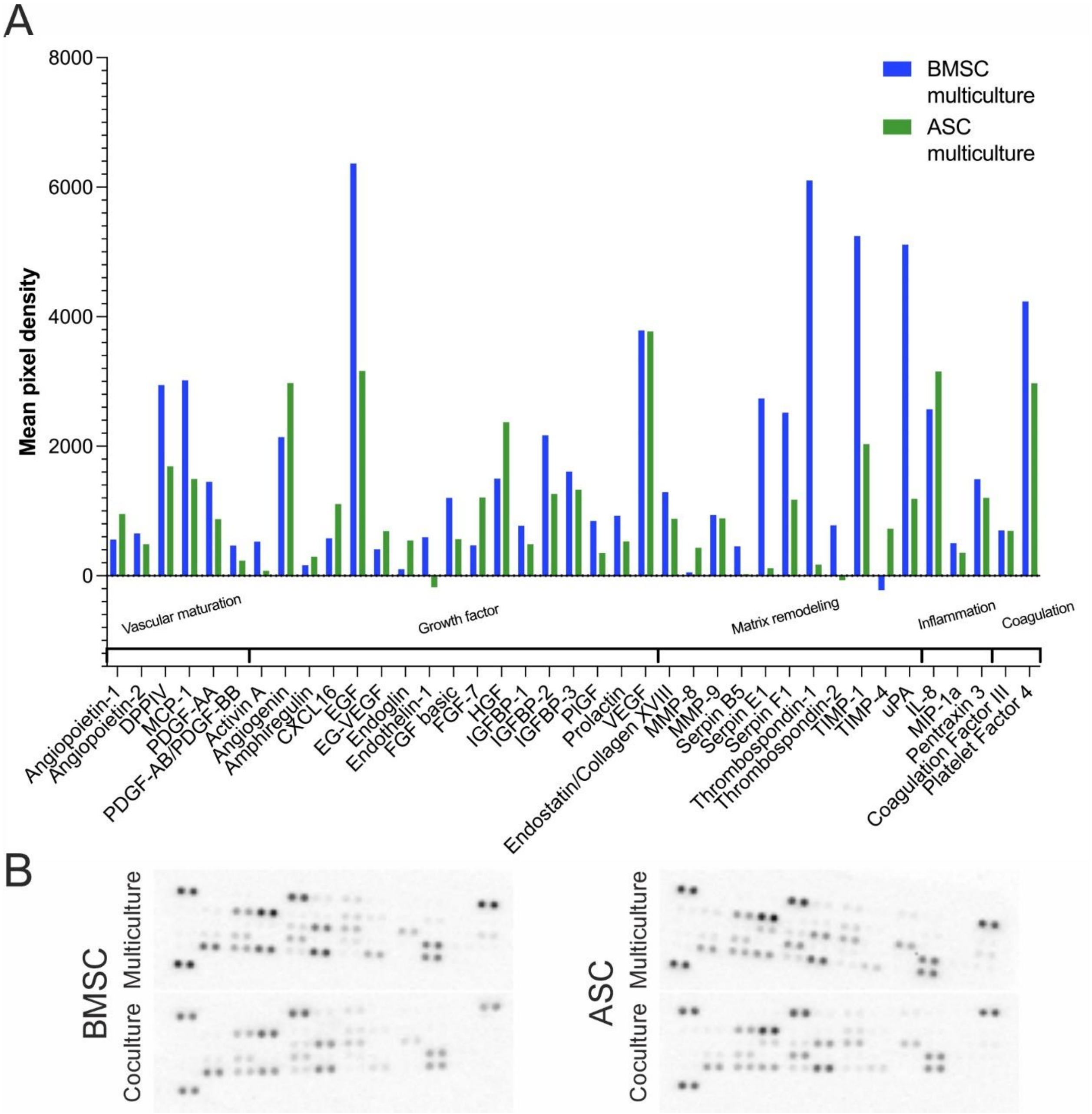

Supplementary Fig. 8 Proteome Profiler human angiogenesis array analysis. a Protein secretion profile of BMSC and ASC multicultures normalized to BMSC and ASC cocultures, respectively. Most of the angiogenesis-related proteins are overexpressed in multicultures compared to cocultures. **b** Proteome Profiler membranes from BMSC and ASC multi- and cocultures used for spot intensity quantification. Medium for analysis was collected from experiment 6. Details of the BMSC and ASC cell lines used in the experiment are listed in Supplementary Tables 1, 2 and 3.

Supplementary Tables

Supplementary Table 1 Donor information for the used primary mesenchymal stem/stromal cell lines BMSC 1-2 and ASC 1-2. For donor cell line BMSC 1, Body Mass Index (BMI) was not available (N/A).

Donor ID	Gender	Age	BMI
**BMSC 1**	Male	82	N/A
**BMSC 2**	Female	87	28.9
**ASC 1**	Male	56	25.2
**ASC 2**	Female	33	29.1

Supplementary Table 2 Surface marker expression of the studied donor BMSCs and ASCs. Individual donor cell line passage during analysis is denoted in the column ''P''. Positive > 80%; low < 10%; negative < 2%. The cells were characterized as MSCs due to positive expression of CD73, CD90, and CD105, and low or negative expression of CD14, CD19, CD45 [67]. The expression of CD34 and HLA-DR was present at variable levels. The heterogeneity in surface marker expression if compared to the ISCT requirements could be explained by changes in cell culturing conditions [68].

		**Surface marker expression**							
		**CD14**	**CD19**	**CD34**	**CD45**	**CD73**	**CD90**	**CD105**	**HLA-DR**
**Donor ID**	**P**	low to negative	low to negative	variable	low to negative	positive	positive	positive	variable
**BMSC 1**	2	1.2	1.2	0.8	1.6	100.0	98.0	100.0	97.1
**BMSC 2**	3	7.4	7.1	3.1	8.8	95.4	90.2	93.6	92.2
**ASC 1**	2	0.5	0.3	37	0.7	96.8	98.1	99.8	1
**ASC 2**	2	0.9	0.8	51.3	3.4	93.3	99.4	99.8	1.2

Supplementary Table 3 Experimental information of the used primary mesenchymal stem/stromal cell lines BMSC 1-2 and ASC 1-2. Experiments were repeated 7 and 5 times for BMSCs and ASCs, respectively.

Experiment ID	BMSC ID	ASC ID
Experiment 1	BMSC 1	ASC 1
Experiment 2	BMSC 2	ASC 1
Experiment 3	BMSC 2	ASC 2
Experiment 4	BMSC 2	-
Experiment 5	BMSC 2	-
Experiment 6	BMSC 2	ASC 2
Experiment 7	BMSC 2	ASC 2

Supplementary Table 4 Proteins analyzed with the Proteome Profiler array.

Activin A	DPPIV/CD26	GDNF	CCL2/MCP-1	CXCL4/PF4
ADAMTS-1	EGF	GM-CSF	CCL3/MIP-1 alpha	PIGF
Angiogenin	EG-VEGF	HB-EGF	MMP-8	Prolactin
Angiopoietin-1	Endoglin/CD105	HGF	MMP-9	Serpin B5/Maspin
Angiopoietin-2	Endostatin/Collagen XVIII	IGFBP-1	NRG1-beta 1	Serpin E1/PAI-1
Angiostatin/ Plasminogen	Endothelin-1	IGFBP-2	Pentraxin 3	Serpin F1/PEDF
Amphiregulin	FGF acidic	IGFBP-3	PD-ECGF	TIMP-1
Artemin	FGF basic	IL-1 beta	PDGF-AA	TIMP-4
CXCL16	FGF-4	LAP (TGF-beta 1)	PDGF-AB/ PDGF-BB	Tissue Factor/Factor III
CXCL8/IL-8	FGF-7/KGF	Leptin	Persephin	Thrombospondin-1
Thrombospondin-2	uPA	Vasohibin	VEGF	VEGF-C
